# Targeting PPARα in the rat valproic acid model of autism: focus on social motivational impairment and sex-related differences

**DOI:** 10.1186/s13229-020-00358-x

**Published:** 2020-07-27

**Authors:** Simona Scheggi, Francesca Guzzi, Giulia Braccagni, Maria Graziella De Montis, Marco Parenti, Carla Gambarana

**Affiliations:** 1 Department Molecular and Developmental Medicine, University of Siena, Via Aldo Moro, 2, Siena, Italy; 2 Department Medicine and Surgery, University of Milano-Bicocca, Monza, Italy

**Keywords:** Autism spectrum disorder, Reward, Dopamine, Valproic acid, Animal models

## Abstract

**Background:**

The social motivational theory of autism spectrum disorder (ASD) focuses on social anhedonia as key causal feature of the impaired peer relationships that characterize ASD patients. ASD prevalence is higher in boys, but increasing evidence suggests underdiagnosis and undertreatment in girls. We showed that stress-induced motivational anhedonia is relieved by repeated treatment with fenofibrate (FBR), a peroxisome proliferator-activated receptor α (PPARα) agonist. Here, we used the valproic acid (VPA) model of ASD in rats to examine male and female phenotypes and assess whether FBR administration from weaning to young adulthood relieved social impairments.

**Methods:**

Male and female rats exposed to saline or VPA at gestational day 12.5 received standard or FBR-enriched diet from postnatal day 21 to 48–53, when behavioral tests and ex vivo neurochemical analyses were performed. Phosphorylation levels of DARPP-32 in response to social and nonsocial cues, as index of dopamine D_1_ receptor activation, levels of expression of PPARα, vesicular glutamatergic and GABAergic transporters, and postsynaptic density protein PSD-95 were analyzed by immunoblotting in selected brain regions.

**Results:**

FBR administration relieved social impairment and perseverative behavior in VPA-exposed male and female rats, but it was only effective on female stereotypies. Dopamine D_1_ receptor signaling triggered by social interaction in the nucleus accumbens shell was blunted in VPA-exposed rats, and it was rescued by FBR treatment only in males. VPA-exposed rats of both sexes exhibited an increased ratio of striatal excitatory over inhibitory synaptic markers that was normalized by FBR treatment.

**Limitations:**

This study did not directly address the extent of motivational deficit in VPA-exposed rats and whether FBR administration restored the likely decreased motivation to operate for social reward. Future studies using operant behavior protocols will address this relevant issue.

**Conclusions:**

The results support the involvement of impaired motivational mechanisms in ASD-like social deficits and suggest the rationale for a possible pharmacological treatment. Moreover, the study highlights sex-related differences in the expression of ASD-like symptoms and their differential responses to FBR treatment.

## Background

Autism spectrum disorder (ASD) defines a complex heterogeneous neurodevelopmental disorder induced by multiple environmental and genetic etiologies characterized by persistent deficits in social communication and interaction, and restricted, repetitive patterns of behavior, interests, or activities [1]. Over the years, different theories have been proposed to explain the social impairment of ASD subjects. According to the traditional view, the underlying cause is a defective cognitive processing of own and peers’ mental states that is necessary to promote peer-to-peer relationships (“theory-of-mind”) [2]. More recently, the social motivational theory has suggested that ASD subjects fail to entertain peer relationships because of the lack of reward feelings from social stimuli (social motivation deficit or social anhedonia) [3]. In support of this theory, a number of neuroimaging, electrophysiological, and neurochemical data have provided evidence for disrupted reward-seeking tendencies in ASD, emphasizing the involvement of the motivational component of reinforcement processing, and the underlying brain circuitry that includes the ventral striatum, and particularly the nucleus accumbens (NAc) [4]. If proven right, this interpretation would offer a novel potential target for the core symptom domain of social impairments that still lacks effective treatments.

We previously reported that a 2-week treatment with fenofibrate (FBR), an agonist of peroxisome proliferator-activated receptor α (PPARα) clinically used to treat hyperlipidemia, reinstates sucrose self-administration in rats that is disrupted upon chronic exposure to unavoidable stress [5]. The pro-motivational effect of FBR is paired with the increased phasic activity of the dopaminergic neurons that project from the mesencephalic ventral tegmental area (VTA) to the NAc, and the dopamine D_1_ receptor-dependent enhanced phosphorylation of the Thr^34^ residue of dopamine and cAMP-regulated phosphoprotein Mr 32,000 (DARPP-32) in the NAc shell (NAcS) [5]. FBR effectiveness to relieve motivational anhedonia in an animal model of depression led us to hypothesize that this drug could also relieve the social impairment in an animal model of ASD if caused by social anhedonia. In addition, the repeated administration of a PPARα agonist decreases repetitive behavior of the BTBR T+ Itpr3*tf*/J mice, a idiopathic model of ASD [6]. Thus, long-term FBR treatment could ease another core symptom of ASD.

Here, utilizing the validated environmental model of ASD induced by prenatal exposure to valproic acid (VPA) [7], we report the behavioral and neurochemical effects of FBR administered to rats from weaning (postnatal day 21, PND 21) to young adulthood (PND 48–53). We focused on the developmental window between weaning and late adolescence since ASD children show early deficits in social motivation that during development lead to socio-cognitive deficits [3, 8], and adolescence is the critical period for the development of high-order cognitive functions [9]. The prevalence of ASD diagnosis in male subjects suggests sex-related phenotypic differences in the clinical presentation [10]. Thus, both female and male rat offspring were screened at young adulthood to ascertain possible sex differences in ASD-like symptoms and responsivity to FBR treatment. The behavioral results and the combined neurochemical findings documenting changes in brain areas involved in the dopaminergic reward circuitry may be regarded as a proof-of-concept of the motivational theory, hence supporting a novel pharmacological strategy for the treatment of ASD social deficits.

## Methods

### Animals

All animal care and experimental procedures complied with guidelines of the European Parliament and Council Directive for Care and Use of Laboratory Animals (2010/63/EU) and the Italian legislation (D.L. 2014/26), and the ARRIVE guidelines. Animal care and experimental protocols were approved by the Italian Ministry of Health (Authorization N. 70/2018-PR).

Experiments were performed in male and female Sprague Dawley rats (Charles River, Calco, Italy) that were housed in groups of 3–4 animals per cage, and kept in an environment at constant temperature (22 ± 2 °C) and humidity (55 ± 10%), on a reverse 12 h light–12 h dark cycle (lights off at 7:00 am, lights on at 7:00 pm), and with free access to food and water. Food deprivation was never applied.

### Generation of the VPA rat model of ASD

For mating, the fertility cycles of females were controlled, and the first day of pregnancy (gestational day 0, G0) was the day when the vaginal plug was found. The ASD model was induced by a single intraperitoneal administration of 500 mg/kg sodium valproate (VPA) in 0.9% (w/v) sodium chloride (saline, 2 ml/kg body weight) on G12.5. Control female rats received an equal volume of saline on G12.5. The effects of in utero VPA exposure change according to the dose and the time window of the exposure [11] and administration of this dose at this gestational age was shown to induce malformations associated with behavioral defects, without a dramatic increase in miscarriages. VPA-treated females and their offspring looked generally healthy, although VPA pups showed minor or more pronounced crooked tails and/or chromodacryorrhea. Mothers were housed individually and allowed to raise their own litters.

In order to compare representative developmental milestones between VPA- and saline-exposed pups, negative geotaxis and olfactory discrimination tests were performed. To assess motor coordination and vestibular sensitivity, pups were evaluated for negative geotaxis daily from PND 7 to PND 12 [7]. Pups were individually placed on a 25° inclined surface in a head down position and the time to complete a 180° upward turn was recorded. The cutoff time was set to 2 min.

In order to study the nest-seeking response mediated by the integration of stimuli originated in the olfactory system, pups were tested from PND 9 to PND 12 inside a Plexiglas cage (20 cm *l* × 8 cm *w* × 8 cm *h*) with a cover containing clean bedding on one side and home bedding on the opposite side [7]. A line marked the center of the cage. Each pup was placed in the center and the latency to reach the home bedding area with the front paws and head was recorded.

### Pharmacological treatment

On PND 21, rats were weaned, separated according to sex, and randomly assigned to four treatment groups: saline-exposed treated with standard diet (saline-SD), saline-exposed treated with fenofibrate (FBR)-enriched diet (saline-FBR), VPA-exposed treated with standard diet (VPA-SD), and VPA-exposed treated with FBR-enriched diet (VPA-FBR). Saline- and VPA-exposed animals were fed the standard (SD) or FBR-enriched diet from weaning to the end of experimental procedures. Body weight and food intake were measured on alternate days to estimate general health, possible differences in diet consumption, and FBR intake (data not shown).

### Behavioral tests

**Fig. 1:**
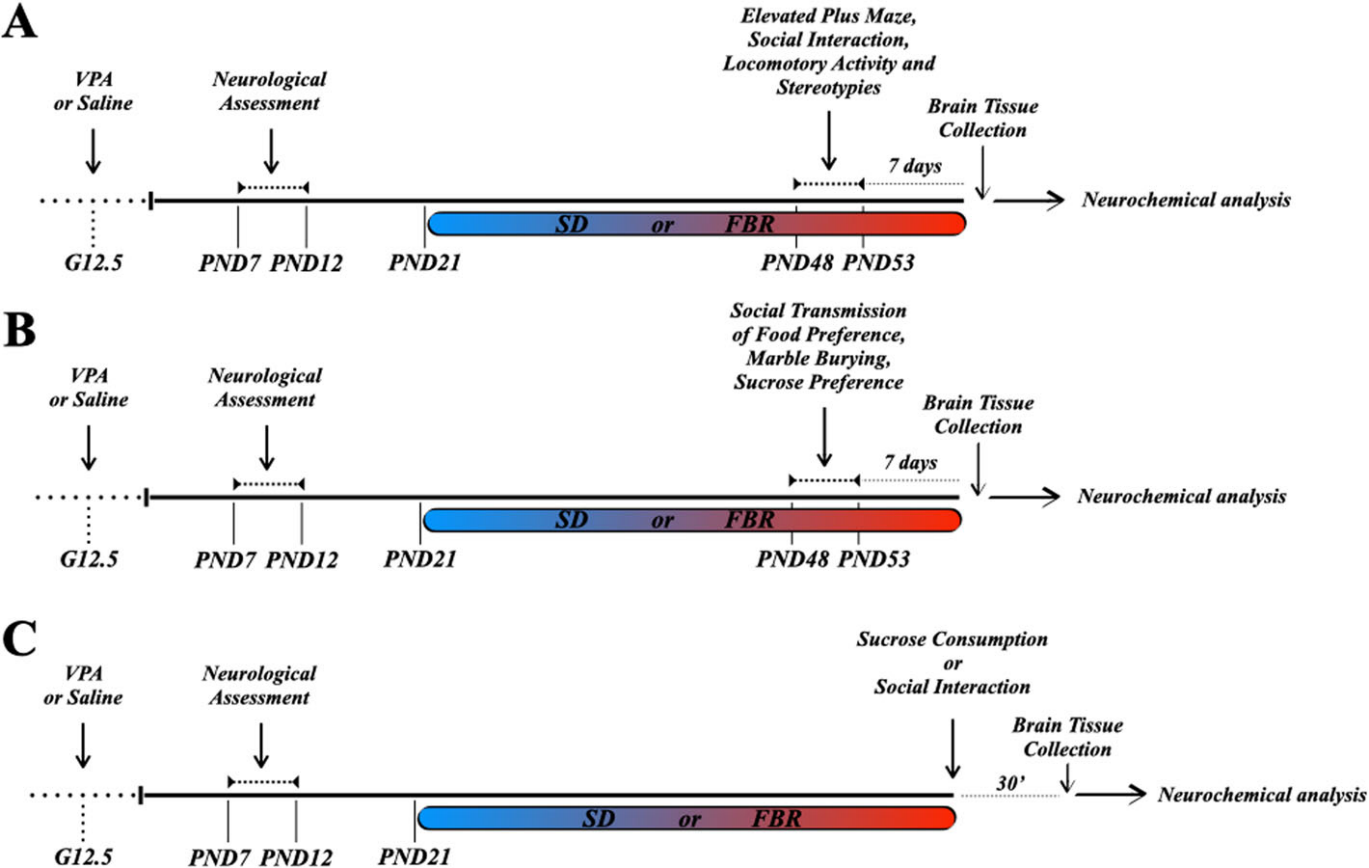
Outline of experimental protocols. ASD-like symptoms were induced in Sprague-Dawley male and female rat offspring of dams that had received an intraperitoneal injection of valproic acid (VPA, 500 mg/kg) at gestational day 12.5 (G 12.5). Offspring were first checked for negative geotaxis and olfactory discrimination (tests of developmental milestones) and from postnatal day (PND) 21 to the end of the experimental procedures were fed with a standard diet (SD) or fenofibrate-enriched diet (FBR). After 4 weeks of treatment, animals were subjected to behavioral and/or neurochemical analysis. **a** From PND 48–53, the four experimental groups (each group *n* = 12) were behaviorally tested to evaluate the level of anxiety (elevated plus maze test), social interaction (three-chamber test), locomotor activity, and stereotypies. One week after the end of behavioral tests, animals were sacrificed and brain regions were dissected out for immunoblotting assays. **b** The four experimental groups (*n* = 12) of second cohort underwent behavioral screening to evaluate social transmission of food preference and perseverative behavior (marble burying test). In addition, animals were tested for the two-bottle sucrose preference as an index of hedonic response. One week later, animals were sacrificed and brain regions were dissected out for immunoblotting assays. **c** The third cohort was used to determine by immunoblotting the Thr^34^ phosphorylation levels of DARPP-32 in response to social interaction or nonsocial stimulus (sucrose consumption) in the shell of NAc (NAcS). At PND 48–53, half animals in each group were sacrificed at baseline and half 30 min after a 10 min-interaction with a novel conspecific (social stimulus) or 30 min after consumption of 10 sucrose pellets. For each experimental group in this cohort, the rats not exposed to the social or sucrose stimulus were also used to assay the PPARα levels in the VTA

The expression of an ASD-like phenotype and the possible effects of treatment were evaluated by behavioral tests performed from PND 48–53 or PND 120 between 09:00 am and 5:00 pm under red light illumination and noise-free condition. From weaning, male and female rats were kept in distinct, identical, and adjacent rooms. One week after the last behavioral test, the first and second cohort were sacrificed for ex vivo neurochemical experiments (Fig. 1a, b). Each rat was exposed to one behavioral test per day on alternate days. The sucrose preference test was performed 2 days after the previous test. The test sequences were as follows: elevated plus maze, social interaction, locomotor activity, and stereotypies assessment (first cohort) and social transmission of food preference, marble burying, and sucrose preference (second cohort) (Fig. 1a, b). Rats were transferred from the housing room to the experimental room 60 min prior to the beginning of each experiment to habituate to the test environment. The third cohort rats were sacrificed after exposure to a social or palatable stimulus (Fig. 1c).

#### Social interaction

Social behavior was assessed by the three-chamber test using a dedicated apparatus (120 cm *l* × 40 × *w* × 40 cm *h*; Ugo Basile, Gemonio, Italy). During the habituation phase on the day before the test, rats had free access to the whole apparatus for 10 min. During the 10-min test, a non-familiar control rat of the same sex was placed inside a cage (social stimulus) in a side chamber and an empty cage was placed in the opposite chamber (nonsocial stimulus). The time spent sniffing and exploring each cage and the numbers and latencies to the first social interaction bout were recorded. Moreover, the sociability index (SI) was calculated as the ratio between the time spent interacting with the social stimulus over the time spent interacting with the nonsocial stimulus.

#### Social transmission of food preference test

The test was adapted from the protocol described by Wrenn [12]. In preliminary experiments, we verified that control and VPA-exposed rats eat the novel flavored food to be used in the test (oven-baked and salty crispy chips, Original Ritz Cracker, Nabisco, East Hanover, NJ, USA), and preferred this food to their standard diet in a 2-h session (data not shown). These animals were not used for the test. Unfamiliar control rats of the same sex of test rats were used as demonstrators. All rats (experimental subjects and demonstrators) were group-housed until the beginning of test and they were never food-deprived or -restricted. On the test day, the demonstrator rat was placed in a separate box in a separate room and exposed to 3.5 g of the novel flavored food in a small cup dish for 10 min. Demonstrators that ate less than 0.5 g of flavored food were not used in the experiment. The demonstrator was then moved to the adjacent test room and placed in a clean cage where the test rat had just been placed. Free interactions were allowed for 10 min. Next, the test rat was transferred to a clean cage, exposed to the novel flavored food in a small cup dish (Fig. 3k), and its latency to eat was recorded. The latency to eat the novel flavored food in the same environment and after the same manipulations used for the test, but without social interaction, was measured in a different set of rats in the four experimental groups (Fig. 3l, m).

#### Marble burying test

Each rat was placed in a clean polycarbonate cage (26 cm *l* × 48 cm *w* × 20 cm *h*) with clean, fresh, and unscented bedding (depth 5 cm), where 20 standard glass toy marbles (assorted colors, 16 mm diameter, 5.7 g in weight) had been arranged on the surface with a 5 × 4 order [13]. The marbles were washed with a mild detergent, rinsed in distilled-deionized water, and dried prior to each use. Individual rats were gently placed always in the same box corner and the number of marbles buried in 20 min was recorded.

#### Locomotor and repetitive/stereotypic-like activity

Locomotor activity was monitored in an apparatus, consisting of eight compartments (40 × 45 × 50 cm) with a transparent Perspex cage (23 × 33 × 19 cm) in each compartment, equipped with infrared sensors to detect horizontal locomotor activity and rearing (Actimètre, Imetronic, Pessac, France). Total motility counts in 30 min were recorded, after a 10-min habituation period. Stereotypies were evaluated by an observer blind to the experimental groups using a modified rating scale ranging from 0 to 6 [14, 15]: 0, asleep or motionless; 1, active; 2, active with intermittent bursts of stereotypies; 3, discontinuous stereotyped sniffing, licking, and grooming; 4, frequent stereotyped grooming, sniffing, and licking; 5, continuous stereotyped grooming and licking; and 6, continuous, intense stereotyped grooming that disrupts gross motility.

#### Sucrose consumption test

Rats were given for 24 h a free choice between two bottles, one containing a 2% (w/v) sucrose solution and another tap water. To prevent a side preference effect in drinking behavior, the position of the two bottles was switched after 12 h. No food or water deprivation was applied before or during the test. The preference for sucrose was calculated as percent volume of sucrose solution over total liquid volume consumed.

#### Elevated plus maze (EPM) test

Anxiety-related behaviors were measured using the EPM test [16, 17]. The apparatus consisted of four arms (10 cm *w* and 50 cm *l*): two opposite open arms and two opposite closed arms equipped with high walls (40 cm). This apparatus was elevated above the floor (60 cm) and kept under a 100-lux light. Rats were individually placed at the center of the maze and allowed to explore the apparatus for 5 min. The maze was carefully cleaned with 30% (v/v) ethanol solution and rinsed with distilled water after each test. The time spent in the open and closed arms was recorded. The percentage of time spent in open or closed arms was calculated by the formula: (time in open or closed arms/time in open + closed arms) × 100.

### Immunoblotting

One week after the last behavioral test, rats in the first and second cohort were sacrificed by decapitation after 2–3-min exposure to 3% isoflurane vapors to comply with the requirements for humane endpoints in animal sacrifice [18]. Rats in the third cohort used to analyze Thr34 DARPP-32 phosphorylation levels were sacrificed without isoflurane exposure, as this may modify the phosphorylation levels of several proteins, included DARPP-32 [19, 20]. Heads were briefly immersed (3–5 s) in liquid nitrogen and brains rapidly removed. The caudate-putamen (CPu), NAcS, and VTA were dissected out from the slices corresponding to plates 10–13 (AP: + 2.20) and 41–43 (AP: − 5.30) of Rat Brain Atlas [21]. An ice-cold brain matrix (ASI Instrument Inc., MI, USA) was used to prepare 1 mm-thick (CPu, NAcS) or 0.5 mm-thick (VTA) coronal sections, and brain regions were dissected by micropunching using a stainless-steel biopsy needle (inner diameter 0.61 mm). Dissected brain areas were then snap frozen in liquid nitrogen. Detailed information on tissue sample preparation, dilution, SDS-PAGE electrophoresis/transfer, and immunoblotting conditions are provided in Additional file 1. Briefly, to analyze the expression of synaptic and receptor proteins, striatal tissues were sonicated in RIPA buffer containing protease inhibitors and prepared as indicated in Additional file 1. After the electrophoresis, membranes were incubated with the following primary antibodies: mouse monoclonal anti-rat PSD-95 (#MA1-045 Thermo Fisher Scientific, Waltham, MA; dilution 1:2000), mouse monoclonal anti-rat vGAT (#131011 Synaptic Systems, Goettingen, Germany; dilution 1:1000), rabbit polyclonal anti-rat vGLUT1 (#135303 Synaptic Systems; dilution 1:30,000), mouse monoclonal anti-rat NR1 (#114011 Synaptic Systems; 1:3000), rabbit monoclonal anti-rat NR2A (#124913 Abcam, Cambridge, UK; dilution 1:1000), goat polyclonal anti-human NR2B (#SC1469 Santa Cruz Biotechnology, Dallas, TX; dilution 1:1000), rabbit polyclonal anti-human GluR1 (#31232 Abcam; 1:500), and rabbit polyclonal anti-rat actin (#A2066 Sigma-Aldrich, Milan, Italy; 1:1000). For the analysis of DARPP-32 phosphorylation levels in the NAcS and PPARα levels in VTA, frozen samples were sonicated in 1% (w/v) SDS and 50 mM NaF containing protease inhibitor cocktail. Samples containing 20–30 μg of total proteins were run onto 4–15% Criterion™ TGX Stain-free™ precast gels (#5678085, Bio-Rad Laboratories) and transferred to nitrocellulose membranes (#1620167, Bio-Rad Laboratories). Stain free™ gel formulation incorporates a trihalo compound that, when exposed to ultraviolet (UV) irradiation, catalyzes a covalent reaction between the trihalo compound and tryptophan residues. The resulting “activated” protein fluorescence under UV excitation can be readily detected by suitable imaging systems either within the gel or after transfer to a blotting membrane [22]. After electrophoresis, gels were activated under UV light using the ChemiDoc^TM^ Touch Imaging System (Bio-Rad Laboratories) and then transferred to a nitrocellulose membrane. Following protein transfer, the fluorescent membrane was detected by UV and blot image was collected for total protein. Membranes were then incubated the following primary antibodies: phospho-Thr^34^ DARPP-32, (rabbit monoclonal #12438, Cell Signaling Technology, Beverly, MA; dilution: 1:1000), DARPP-32 (rabbit polyclonal #2302, Cell Signaling Technology; dilution: 1:1000), and PPARα (rabbit polyclonal #SAB 4502260, Sigma-Aldrich; dilution 1:1000). Membranes incubated with anti-phospho-Thr^34^ DARPP-32 antibodies were stripped and re-probed with anti-DARPP-32, and eventually stripped and re-probed with mouse monoclonal anti-β-actin antibody (*#A1978*, *Sigma-Aldrich*; dilution 1:5000) to control for equal loadings. Blots incubated with the anti-PPARα antibody were stripped and re-probed using anti-β-*actin.* Finally, blots were washed as above and chemiluminescence was detected and quantified with the ChemiDoc^TM^ XRS^+^ Imaging System using the Clarity Western ECL substrate (#1705061, Bio-Rad Laboratories).

### Drugs

Sodium valproate (PubChem CID 16760703) was purchased from Sigma-Aldrich and FBR (PubMed CID 3339) was obtained from Fisher Scientific Italia (Rodano, Italy). According to previous studies [5, 23, 24], the FBR diet was a custom-prepared rodent diet (4RF21) enriched with 0.2% FBR (w/w) (Mucedola, Settimo Milanese, Italy), resulting in an estimated average intake of 200 mg/kg/day.

### Data and statistical analysis

All data are expressed as means ± SEM and analyses were performed using GraphPad Prism 7 statistical package (GraphPad, San Diego, CA, USA). Group sizes (*n*) for all experiments are provided and refer to independent single measurements. The results of neurological tests were analyzed by repeated measures (RM) ANOVA with VPA exposure as between factor and time as within factor. Behavioral and neurochemical data were analyzed by two-way ANOVA with VPA exposure and treatment (FBR-enriched or SD diets) as factors. In the immunoblotting experiments, the values from treatment groups were normalized to the corresponding control values and expressed as percentages. Results from DARPP-32 phosphorylation assays were subjected to three-way ANOVA with VPA exposure, treatment, and social interaction/sucrose consumption as factors. Post hoc analysis was performed by the Bonferroni’s test when *p* < 0.05 for the interaction between the factors VPA exposure and FBR treatment (two-way and three-way ANOVA), or for the factors VPA exposure and time (RM ANOVA). Group size was determined by power analysis calculated using the variance estimates obtained from pilot experiments. The experimenters were blind to the treatments and, whenever possible, to saline or VPA prenatal exposure (tail malformations or chromodacryorrhea sometimes revealed VPA exposure).

## Results

### Generation of the VPA rat model of ASD

**Fig. 2:**
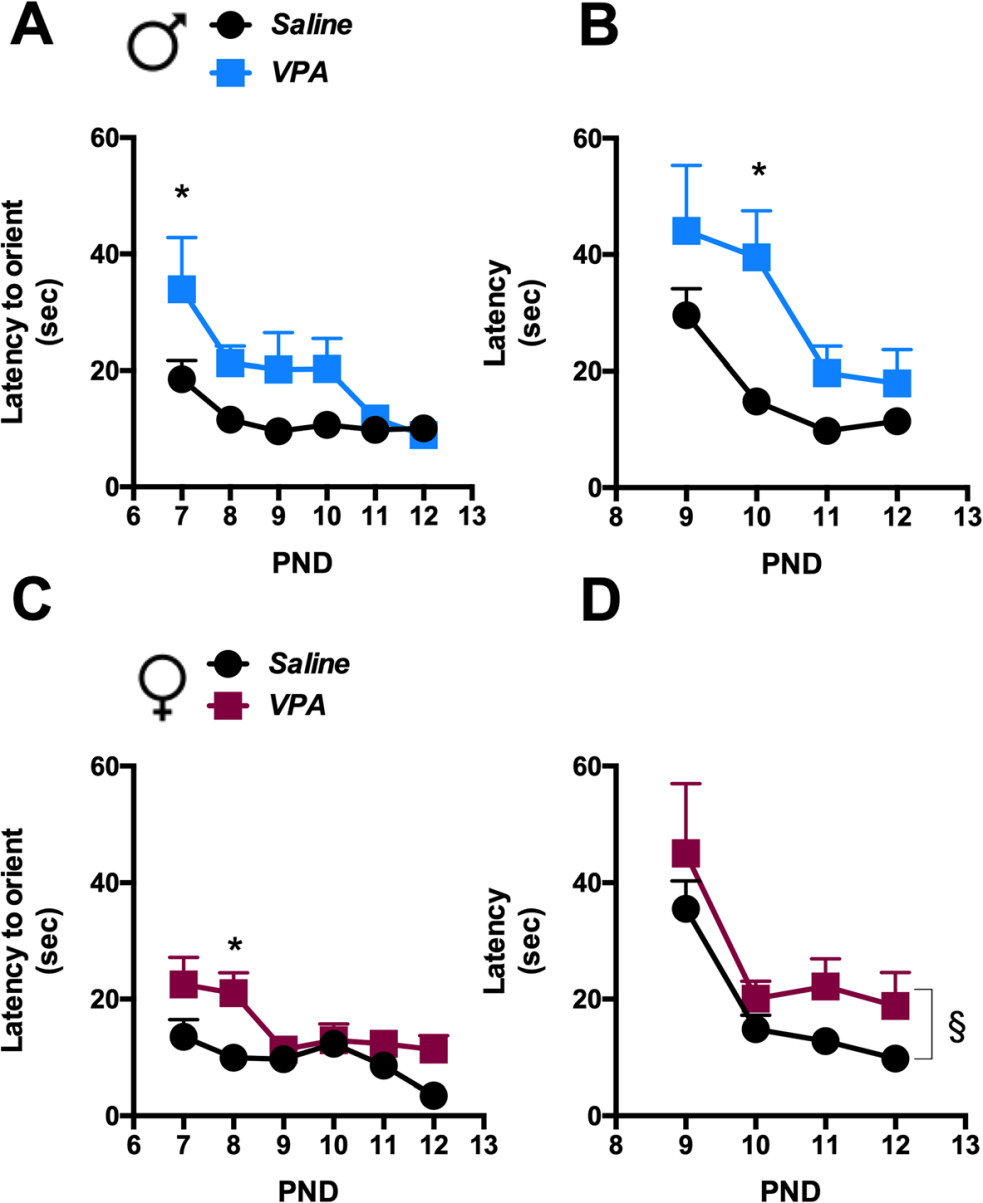
VPA exposed male and female offspring exhibited neurodevelopmental delay*.* Negative geotaxis was evaluated daily in male (a) and female (c) rats from PND 7 through 12. Pups were individually placed on a 25° inclined surface in a head down position and the time to complete a 180° upward turn was recorded. VPA-exposure caused a delayed turning ability in pups of both sexes. a Two-way ANOVA, VPA exposure: *F*_1, 22_ = 7.94, *p* = 0.01; time: *F*_5, 110_ = 4.99, *p* = 0.0004; interaction: *F*_5, 110_ = 1.34, n.s.; post hoc comparison: *p < 0.05 vs. saline group at PND 7. c Two-way ANOVA, VPA exposure: *F*_1, 22_ = 8.96, *p* = 0.0067; time: *F*_5, 110_ = 5.49, *p* = 0.0001; interaction: *F*_5, 110_ = 1.70, n.s.; post hoc comparison: *p < 0.05 vs. saline group at PND8. Values are expressed as means ± SEM; *n* = 12. The olfactory discrimination test was performed daily in male b and female d rats from PND 9 through 12. Male and female VPA-exposed pups showed a higher latency to reach the home bedding area. b Two-way ANOVA, VPA exposure: *F*_1,22_ = 5.57, *p* = 0.0274; time: *F*_3, 66_ = 10.18, *p* < 0.0001; interaction: *F*_3,66_ = 1.34, n.s.; post hoc comparison: *p < 0.05 vs. saline group at PND 10. d Two-way ANOVA, VPA exposure: *F*_1, 22_ = 4.33, *p* = 0.0492; time: *F*_3,66_ = 9.67, *p* < 0.0001; interaction: *F*_3, 66_ = 0.070, n.s.; ^§^*p* < 0.05 main effect of VPA exposure. Values are expressed as means ± SEM; *n* = 12

Newborns from VPA-treated mothers appeared generally healthy without any gross behavioral modifications, although male and female individuals showed tail malformations and/or chromodacryorrhea [11]. To verify whether VPA rats exhibited any signs of neurological developmental delay as reported in previous studies [7, 25], the ability of saline- and VPA-exposed pups to complete a 180° upward turn on a 25° inclined surface (negative geotaxis test), and to discriminate between fresh and 3-day-old home cage bedding and orient toward the nest stimulus were investigated. Our results confirm the developmental delay in VPA-exposed versus control pups of both sexes (Fig. 2a–d).

### Behavioral effects of FBR administration to ASD-like rats

**Fig. 3:**
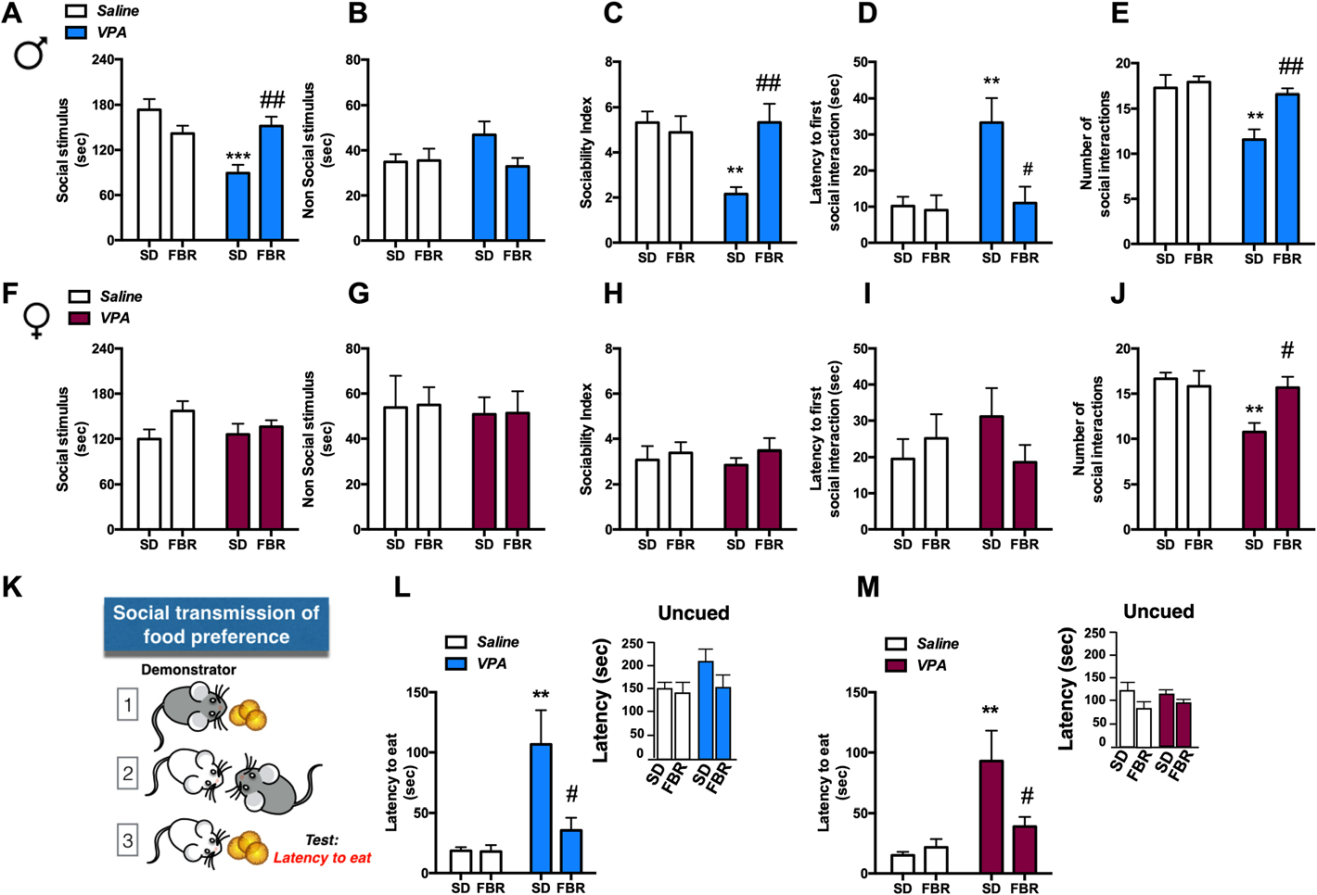
Repeated fenofibrate administration relieved social deficits in young adult rats of both sexes prenatally exposed to VPA*.* The three-chamber test was employed to evaluate the social behavior of male and female rats that had been prenatally exposed to VPA or saline and postnatally treated with FBR or SD. The time spent exploring the social stimulus (**a**, **f**), the nonsocial stimulus (**b**, **g**), the sociability index (SI) (**c**, **h**), the latency to the first bout of social interactions (**d**, **i**), and the number of social interactions (**e**, **j**) were scored. **a** Two-way ANOVA, VPA exposure: *F*_1, 44_ = 9.35, *p* = 0.0038; FBR administration: *F*_1, 44_ = 1.63, n.s.; interaction: *F*_1, 44_ = 15.08, *p* = 0.0003; post hoc comparison: ***p < 0.001 vs. saline-SD group; ^##^*p* < 0.01 vs. VPA-SD group. **b** Two-way ANOVA, VPA exposure: *F*_1, 44_ = 1.008, *p* = n.s.; FBR administration: *F*_1, 44_ = 2.014 n.s.; interaction: *F*_1, 44_ = 2.41, n.s. **c** Two-way ANOVA, VPA exposure: *F*_1, 44_ = 4.84, *p* = 0.033; FBR administration: *F*_1, 44_ = 4.90, *p* = 0.0321; interaction: *F*_1, 44_ = 8.43, *p* = 0.0057; post hoc comparison: **p < 0.01 vs. saline-SD group; ^##^*p* < 0.01 vs. VPA-SD group. **d** Two-way ANOVA, VPA exposure: *F*_1, 44_ = 6.97, *p* = 0.0114; FBR administration: *F*_1, 44_ = 6.07, *p* = 0.017; interaction: *F*_1, 44_ = 5.004, *p* = 0.0304; post hoc comparison: **p < 0.01 vs. saline-SD group; ^##^*p* < 0.01 vs. VPA-SD group. **e** Two-way ANOVA, VPA exposure: *F*_1, 44_ = 11.87, *p* = 0.0013; FBR administration: *F*_1, 44_ = 7.77, n.s.; interaction: *F*_1, 44_ = 4.54, *p* = 0.00386; post hoc comparison: **p < 0.01 vs. saline-SD group; ^##^*p* < 0.01 vs. VPA-SD group. **f** Two-way ANOVA, VPA exposure: *F*_1, 44_ = 0.35, n.s.; FBR administration: *F*_1, 44_ = 3.74, n.s.; interaction: *F*_1, 44_ = 1.21, n.s. **g** Two-way ANOVA, VPA exposure: *F*_1, 44_ = 0.106, n.s.; FBR administration: *F*_1, 44_ = 0.0074, n.s.; interaction: *F*_1, 44_ = 0.0008, n.s. **h** Two-way ANOVA, VPA exposure: *F*_1, 44_ = 0.021, n.s.; FBR administration: *F*_1, 44_ = 0.93, n.s.; interaction: *F*_1, 44_ = 0.106, n.s. **i** Two-way ANOVA, VPA exposure: *F*_1, 44_ = 0.17, n.s.; FBR administration: *F*_1, 44_ = 0.31, n.s.; interaction: *F*_1, 44_ = 2.10, n.s. **j** Two-way ANOVA, VPA exposure: *F*_1, 44_ = 6.35, *p* = 0.0154; FBR administration: *F*_1, 44_ = 2.86, n.s.; interaction: *F*_1, 44_ = 5.67, *p* = 0.0216; post hoc comparison: **p < 0.01 vs. saline-SD group; ^#^*p* < 0.05 vs. VPA-SD group. Values are expressed as mean ± SEM; *n* = 12. **k** The social transmission of food preference test was performed to determine the ability of VPA/saline male and female rats treated with SD or FBR to eat a novel food upon a safety signal transmitted from a conspecific rat that had previously tasted the food. The latency to eat the novel food by male (**l**) and female rats (**m**) was measured. **l** Main panel: two-way ANOVA, VPA exposure: *F*_1, 44_ = 11.73, *p* = 0.0013; FBR administration: *F*_1, 44_ = 5.39, *p* = 0.0248; interaction: *F*_1, 44_ = 5.22, *p* = 0.0272; post hoc comparison: **p < 0.01 vs. saline-SD group; ^#^*p* < 0.05 vs. VPA-SD group. Inset: the latency to eat the novel food without interaction with a demonstrator (uncued) is shown. Two-way ANOVA, VPA exposure: *F*_1, 20_ = 0.91, n.s.; FBR administration: *F*_1, 20_ = 1.66, n.s.; interaction: *F*_1, 20_ = 0.879, n.s. **m** Main panel: Two-way ANOVA, VPA exposure: *F*_1, 44_ = 11.92, *p* = 0.0012; FBR administration: *F*_1, 44_ = 2.95, n.s.; interaction: *F*_1, 44_ = 4.84, *p* = 0.033; post hoc comparison: **p < 0.01 vs. saline-SD group ^#^*p* < 0.05 vs. VPA-SD group. Inset: the latency to eat the novel food without interaction with a demonstrator (uncued) is shown. Two-way ANOVA, VPA exposure: *F*_1, 20_ = 0.202, n.s.; FBR administration: *F*_1, 20_ = 2.99, n.s.; interaction: *F*_1, 20_ = 1.018, n.s. Values are expressed as mean ± SEM; *n* = 12; insets: *n* = 6

In order to verify the presence of social impairments in the VPA offspring and the effects of FBR treatment, we firstly employed the three-chamber test. Male VPA rats spent less time sniffing and exploring the conspecific animal stimulus (Fig. 3a), whereas they spent a similar time exploring the nonsocial stimulus (Fig. 3b), thus showing a reduced sociability index compared to control rats (Fig. 3c). Moreover, they had longer latencies to the first bout of social interactions (Fig. 3d) and lower numbers of social interactions (Fig. 3e). Female VPA rats exhibited milder social deficits in the three-chamber test, as only the number of social interactions was significantly reduced (Fig. 3j), whereas the time spent exploring the social and nonsocial stimulus, sociability index, and latency to first social interaction bout were similar to those of the control group (Fig. 3f–i). FBR administration rescued the impaired social interactions in male and female VPA rats (Fig. 3a, c, d, e, j). Similar results were observed in adult male rats after a 14-week FBR administration (Additional file 2: Figure S1A-C). When we tested the social transmission of food preference (Fig. 3k), both male and female VPA rats exhibited increased latencies to taste a novel food whose safety had been “suggested” by rat demonstrators that had previously consumed the food, and FBR administration reinstated the social transmission of food safety signals (Fig. 3l, m). The longer latencies of rats in all groups to consume the novel food without interaction with demonstrators indicated that the test is an index of social communication skills more than the effects of novel food palatability (Fig. l, m, insets).

**Fig. 4:**
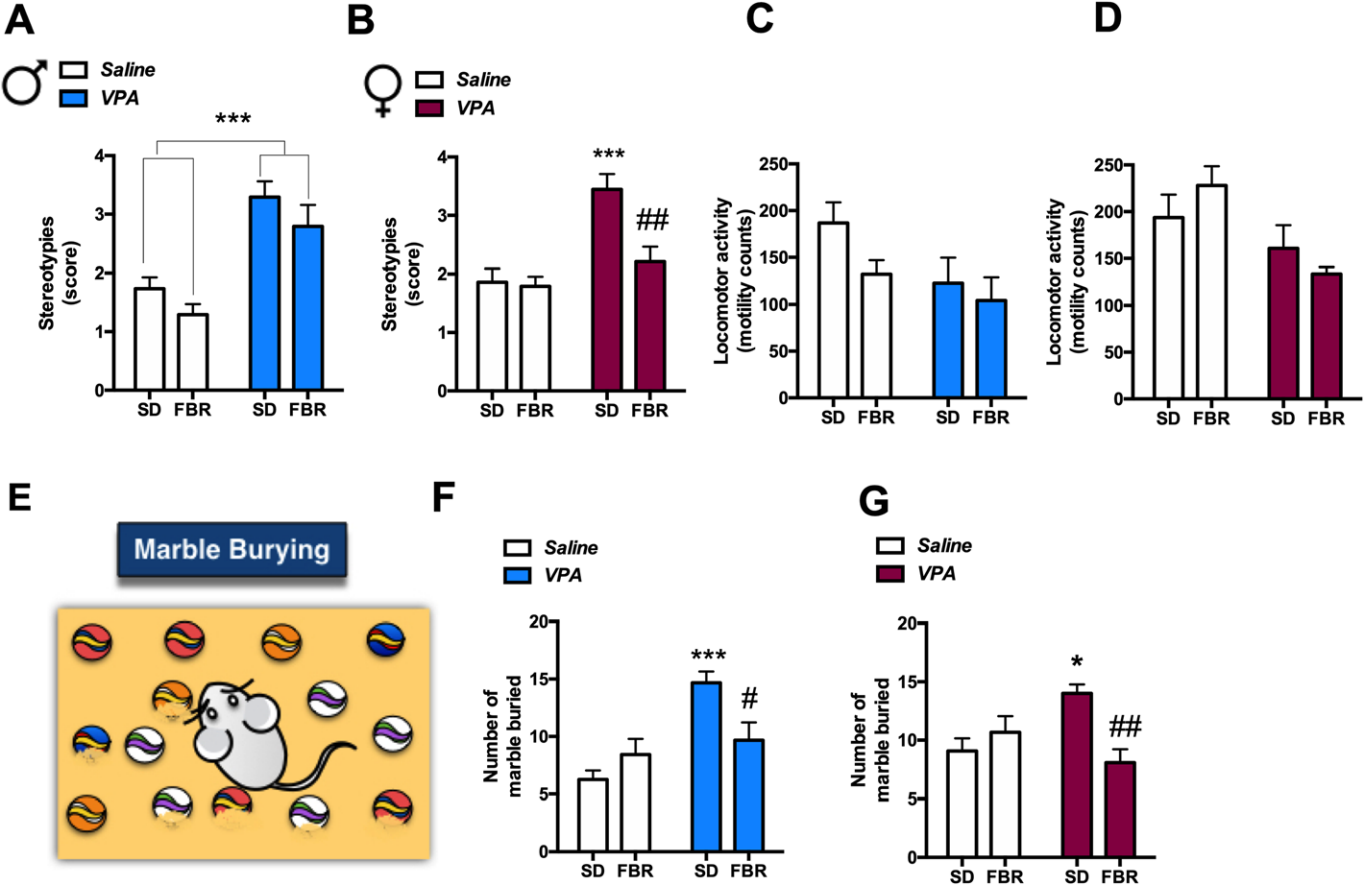
Fenofibrate treatment decreased repetitive behavior of VPA females and perseverative behavior of VPA rats of both sexes*.* Stereotyped movements (self-grooming and lickings) and locomotor activities (**c**, **d**) were examined in VPA and saline male and female rats treated with SD or FBR. **a** Two-way ANOVA, VPA exposure: *F*_1, 44_ = 33.73, *p* < 0.0001; FBR administration: *F*_1, 44_ = 3.22, n.s.; interaction: *F*_1, 44_ = 0.011, n.s. **b** Two-way ANOVA, VPA exposure: *F*_1, 44_ = 18.32, *p* < 0.0001; FBR administration: *F*_1, 44_ = 7.80, *p* = 0.0077; interaction: *F*_1, 44_ = 6.23, *p* = 0.0163; post hoc comparison: ***p < 0.001 vs. saline-SD group; ^##^*p* < 0.01 vs. VPA-SD. **c** Two-way ANOVA, VPA exposure: *F*_1, 44_ = 4.14, *p* = 0.0479; FBR administration: *F*_1, 44_ = 2.61, n.s*.*; interaction: *F*_1, 44_ = 0.63, n.s. **d** Two-way ANOVA, VPA exposure: *F*_1, 44_ = 9.52, *p* = 0.0035; FBR administration: *F*_1, 44_ = 0.028, n.s*.*; interaction: *F*_1, 44_ = 2.23, n.s*.* Values are expressed as mean ± SEM; *n* = 12. Marble burying activity test (**e**) was employed to estimate the effects of prenatal VPA exposure and postnatal FBR treatment on perseverative behavior (**e**) of male (**f**) and female rats (**g**). **F** Two-way ANOVA, VPA exposure: *F*_1, 44_ = 15.80, *p* = 0.0003; FBR administration: *F*_1, 44_ = 1.35, n.s.; interaction: *F*_1,44_ = 8.68, *p* = 0.0051; post hoc comparison: ***p < 0.001 vs. saline-SD group; ^#^*p* < 0.05 vs. VPA-SD group. **g** Two-way ANOVA, VPA exposure: *F*_1, 44_ = 1.08, n.s.; FBR administration: *F*_1, 44_ = 3.74, n.s.; interaction: *F*_1,44_ = 11.21, *p* = 0.0017; post hoc comparison: *p < 0.05 vs. saline-SD group; ^##^*p* < 0.01 vs. VPA-SD group. Values are expressed as mean ± SEM; *n* = 12

Increased stereotyped and perseverative behaviors were detected in male and female VPA rats (Fig. 4), as previously reported [26, 27]. However, we observed a clear sex difference upon FBR administration. While the treatment was ineffective in males (Fig. 4a), it was successful in females (Fig. 4b). When perseverative behavior was evaluated using the marble burying test, both male and female VPA rats exhibited increased burying behavior compared to control rats, and FBR administration reduced this behavior in both sexes (Fig. 4f, g). Long-term FBR administration decreased perseverative behavior also in adult male rats (Additional file 2: Figure S1D). Spontaneous locomotor activity, tested as a possible confounding factor, was not significantly modified by VPA exposure or FBR administration in both sexes (Fig. 4c, d).

**Fig. 5:**
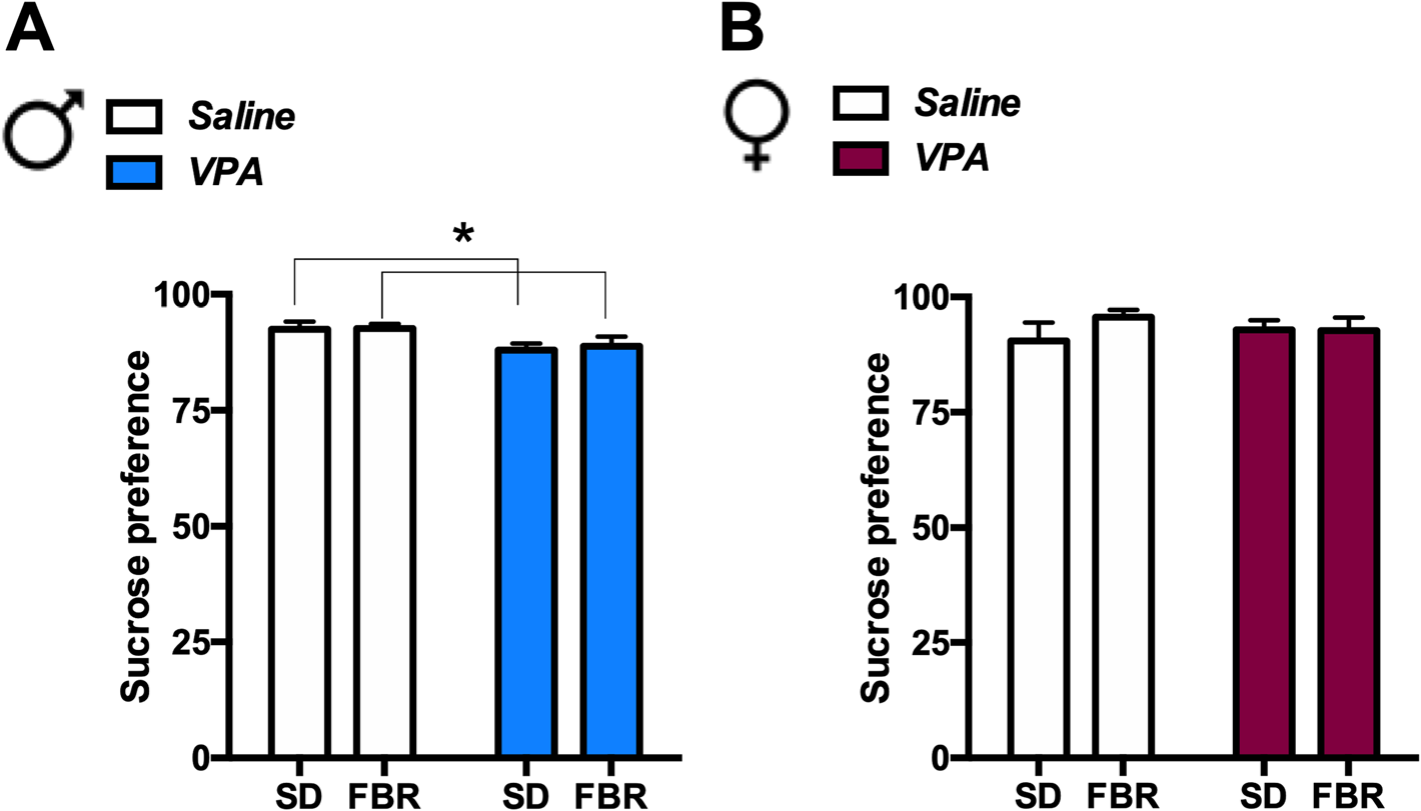
VPA exposure differently affected sucrose preference in male and female rats*.* VPA-exposed male rats, independently from treatment, showed a lower preference for a 2% (w/v) sucrose solution. **a** Two-way ANOVA of sucrose preference expressed as percent volume of sucrose solution over total fluid volume (sucrose solution + water) drunk: VPA exposure: *F*_1, 44_ = 6.77, *p* = 0.0125; FBR administration: *F*_1, 44_ = 0.09, n.s.; interaction: *F*_1, 44_ = 0.045, n.s.). **b** In female rats, no significant effects of VPA exposure, FBR treatment, or their interaction were detected (VPA exposure: *F*_1, 44_ = 0.0044, n.s.; FBR administration: *F*_1, 44_ = 0.81, n.s.; interaction: *F*_1, 44_ = 0.89, n.s.). Values are expressed as mean ± SEM; *n* = 12. *p < 0.05 main effect of VPA exposure

To evaluate the hedonic response, the preference for a 2% (w/v) sucrose solution versus water was determined. VPA-exposed male rats showed a reduced sucrose preference that was unaffected by FBR administration (Fig. 5a). VPA females did not show a decreased hedonic response in the test (Fig. 5b).

**Fig. 6:**
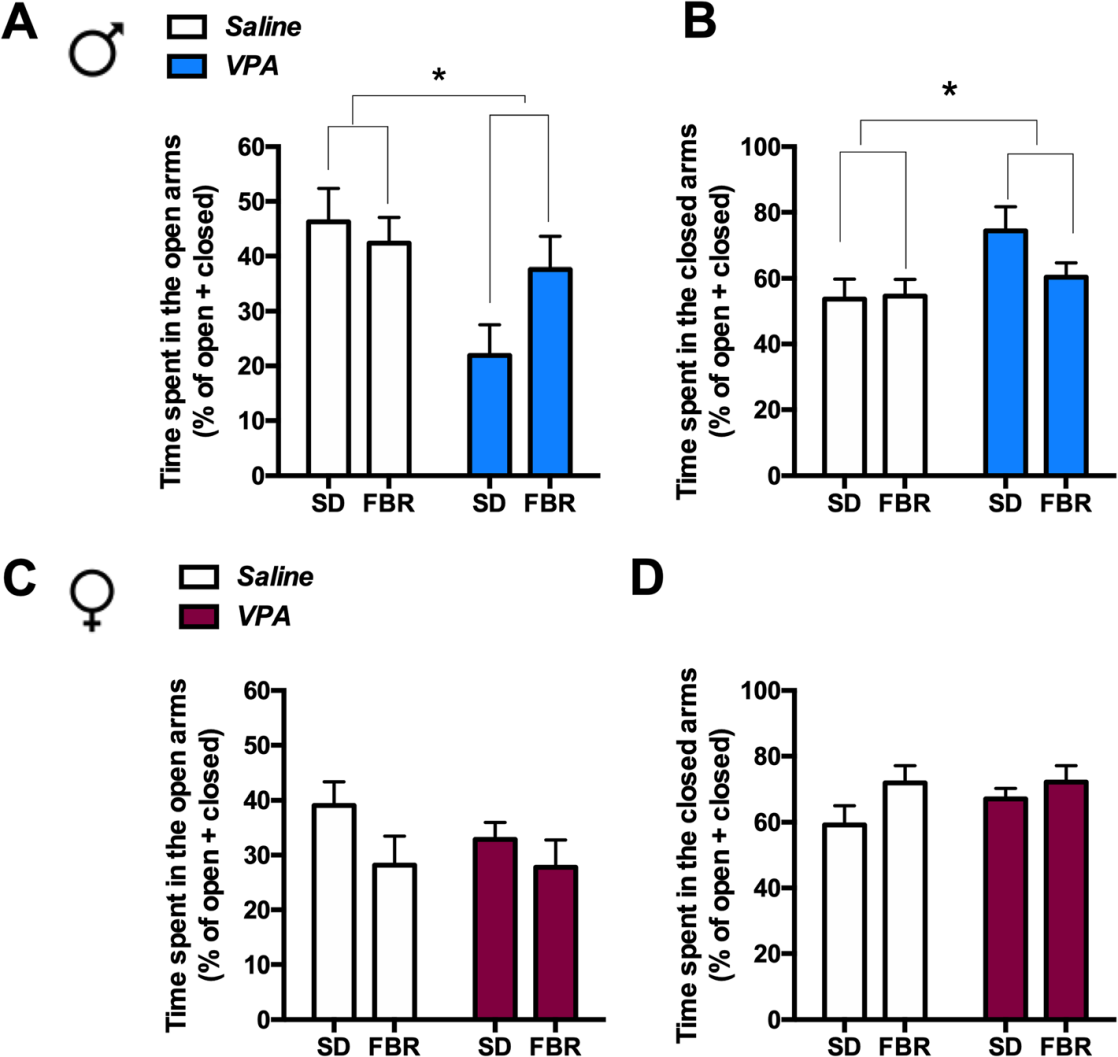
VPA-exposed male rats showed increased anxiety in the elevated plus maze test*.* VPA-exposed male rats showed a reduction of the percentage of time spent in the open arm (**a**) and an increase of the percentage of time spent in the closed arm (**b**). **a** Two-way ANOVA, VPA exposure: *F*_1, 44_ = 6.69, p = 0.013; FBR administration: *F*_1, 44_ = 1.09, n.s.; interaction: *F*_1,44_ = 3.02, n.s. **b** Two-way ANOVA, VPA exposure: *F*_1, 44_ = 4.41, *p* = 0.0413; FBR administration: *F*_1, 44_ = 1.46, n.s.; interaction: *F*_1,44_ = 0.21, n.s. Values are expressed as mean ± SEM of the percentage of time spent in the open or closed arm divided by the total time in open + closed arms; *n* = 12. VPA exposure did not affect anxiety behavior in female rats (**c**, **d**). **c** Two-way ANOVA, VPA exposure: *F*_1, 43_ = 0.54, n.s.; FBR administration *F*_1, 43_ = 3.21, n.s.; interaction: *F*_1,43_ = 0.42, n.s. **d** Two-way ANOVA, VPA exposure: *F*_1, 43_ = 0.69, n.s.; FBR administration: *F*_1, 43_ = 3.21, n.s.; interaction: *F*_1,43_ = 0.59, n.s. Values are expressed as mean ± SEM of the percentage of time spent in the open or closed arm divided by the total time in open + closed arms; *n* = 12. *p < 0.05 main effect of VPA exposure

Finally, the level of anxiety in VPA-exposed rats treated or not with FBR was determined with the EPM test (Fig. 6). VPA males, but not females, were more anxious than control rats spending less time in the open arms and longer time in the safer closed arms of the maze, and this behavior was not modified by FBR treatment (Fig. 6a, b).

### Neurochemical effects of FBR administration in ASD-like rats

**Fig. 7:**
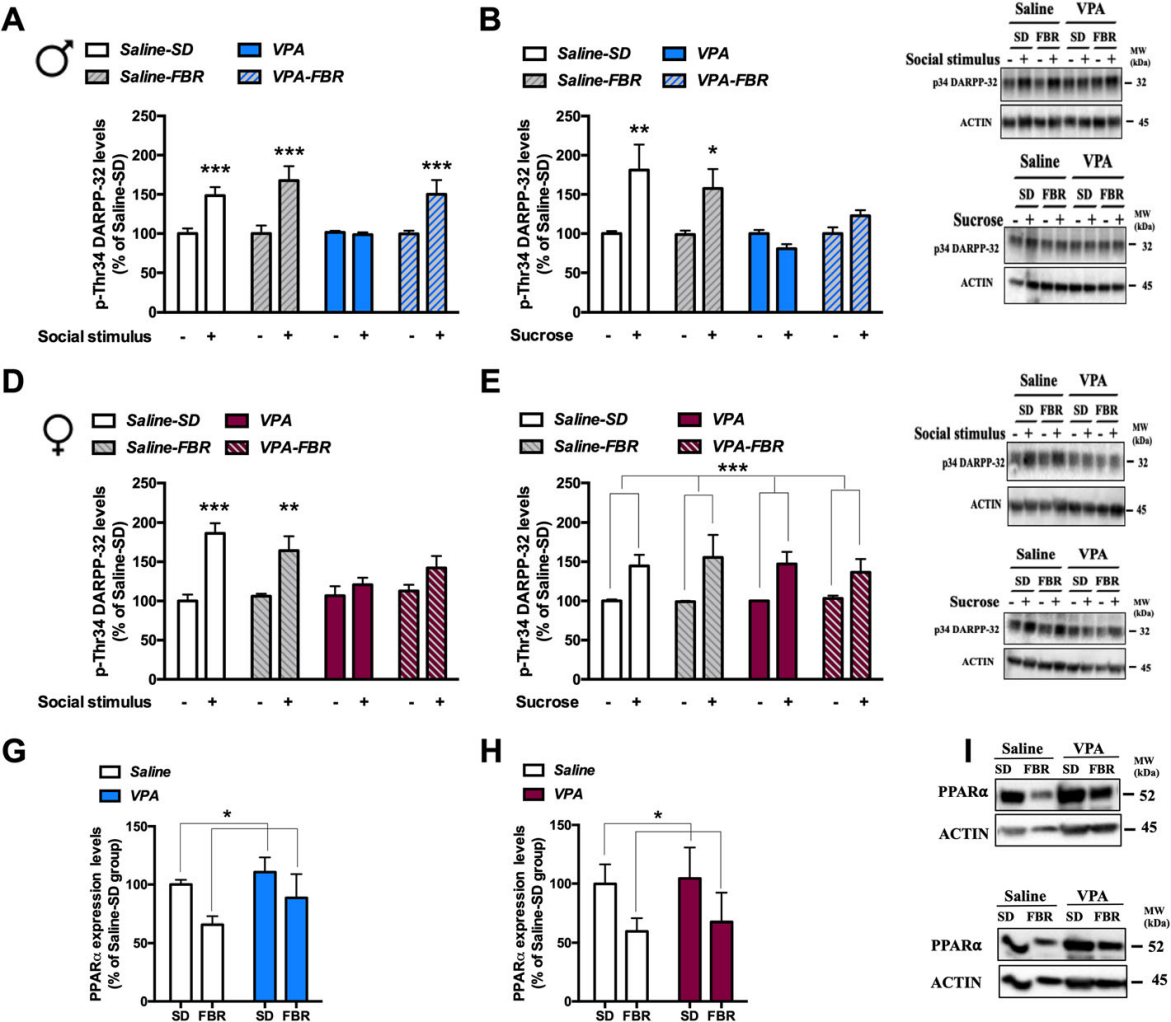
Fenofibrate administration rescued the NAcS dopaminergic response to rewarding social stimulus in male rats blunted by VPA exposure*.* The levels of Thr^34^ DARPP-32 phosphorylation at baseline and following the interaction with an unknown conspecific or after sucrose consumption were measured in the NAcS by immunoblotting in male (**a**, **b**) and female rats (**d**, **e**). **a** Three-way ANOVA, social stimulus: *F*_1,40_ = 72.13, *p* < 0.0001; VPA exposure: *F*_1,40_ = 11.59, *p* = 0.0015; FBR administration: *F*_1,40_ = 10.70, *p* = 0.022; interaction of VPA exposure × social stimulus: *F*_1,40_ = 11.82, *p* = 0.0014; interaction of FBR administration × social stimulus: *F*_1,40_ = 16.02, *p* = 0.0003; interaction VPA exposure × FBR administration × social stimulus: *F*_1,40_ = 4.109, *p* = 0.0494; post hoc comparison: ***p < 0.001 vs. the respective baseline group. Values are expressed as mean ± SEM and calculated as percentage of the baseline values of the saline-SD group; *n* = 6. **b** Three-way ANOVA, sucrose consumption: *F*_1,40_ = 11.86, *p* = 0.0014; VPA exposure: *F*_1,40_ = 11.83, *p* = 0.0014; FBR administration: *F*_1,40_ = 0.36, n.s.; interaction of VPA exposure × FBR administration: *F*_1,40_ = 2.19, n.s.; interaction of VPA exposure × sucrose consumption: *F*_1,40_ = 12.68, *p* = 0.0010; interaction of VPA exposure × FBR treatment × sucrose consumption: *F*_1,40_ = 2.57, n.s; post hoc comparison: **p < 0.01, *p < 0.05 vs. the respective baseline group. Values are expressed as mean ± SEM and calculated as percentage of the baseline values of the saline-SD group; *n* = 6. **c** Representative immunoblotting in males. **d** Three-way ANOVA, social stimulus: *F*_1,40_ = 48.48, *p* < 0.0001; VPA exposure: *F*_1,40_ = 7.59, *p* = 0.0088; FBR administration: *F*_1,40_ = 0.19, n.s.; interaction of VPA exposure × social stimulus: *F*_1,40_ = 14.12, *p* = 0.0005; interaction of VPA exposure × FBR administration: *F*_1,40_ = 2.51, n.s.; interaction of VPA exposure × FBR treatment × social stimulus: *F*_1,40_ = 2.52, n.s.; post hoc comparison: ***p < 0.001, **p < 0.01 vs. the respective baseline group. Values are expressed as mean ± SEM and calculated as percentage of the baseline values of the saline-SD group; *n* = 6. **e** Three-way ANOVA, sucrose consumption: *F*_1,32_ = 35.08, *p* < 0.0001; VPA exposure: *F*_1,32_ = 0.15, n.s*;* FBR administration: *F*_1,32_ = 0.007, n.s.; interaction of VPA exposure x FBR administration: *F*_1,40_ = 2.19, n.s.; interaction of VPA exposure × sucrose consumption: *F*_1,32_ = 0.45, n.s*.*; interaction of VPA exposure × FBR treatment × sucrose consumption: *F*_1,32_ = 0.69, n.s. Values are expressed as mean ± SEM and calculated as percentage of the baseline values of the saline-SD group; *n* = 5. **f** Representative immunoblotting in females. Values are expressed as mean ± SEM and calculated as percentage of the baseline values of the saline-SD group. The levels of PPARα were measured by immunoblotting in the VTA of male (**g**) and female rats from the subgroups not exposed to the social or sucrose stimulus (**h**). **g** Two-way ANOVA, VPA exposure: *F*_1,24_ = 1.75, n.s.; FBR administration: *F*_1,24_ = 4.97, *p* = 0.0354; interaction: *F*_1,24_ = 0.226, n.s. Values are expressed as mean ± SEM and calculated as percentage of the saline-SD group values; *n* = 7. **h** Two-way ANOVA, VPA exposure: *F*_1,24_ = 0.176, n.s.; FBR administration: *F*_1,24_ = 4.39, *p* < 0.049; interaction: *F*_1,24_ = 0.00024, n.s. **i** Representative immunoblotting in males (upper panel) and females (lower panel). Values are expressed as mean ± SEM and calculated as percentage of the saline-SD group values; *n* = 7

In order to study the neurochemical mechanisms underpinning the impairment in social behavior exhibited by VPA rats and the effects of FBR administration, we focused on the NAcS, a key brain region in the neural circuitry mediating the motivational component of reward behavior [28, 29]. The dopamine D_1_ receptor-mediated phosphorylation of the Thr^34^ residue of DARPP-32 by protein kinase A in the NAcS can be considered an index of the activation of the dopaminergic mesolimbic pathway in response to salient cues [30]. Accordingly, a reduced behavioral response to a rewarding stimulus, such as palatable food, correlates with a blunted dopamine D_1_ receptor-mediated response in the NAcS [5, 30, 31]. Thus, to test the hypothesis that social deficits in the VPA model correlate with impaired motivation [3] and a blunted dopaminergic response, we assayed by immunoblotting the phosphorylation levels of the Thr^34^ residue of DARPP-32 (p-Thr^34^ DARPP-32) in the NAcS at baseline and 30 min after a 10-min interaction with an unknown control rat of the same sex. Levels of p-Thr^34^ DARPP-32 increased after the social interaction in the male and female control groups fed with the standard or FBR-enriched diets (Fig. 7a, d). In contrast, the stimulus-induced increase in p-Thr^34^ DARPP-32 levels was blunted in VPA-exposed rats of both sexes, but this response was rescued by FBR administration in male rats only (Fig. 7a, d). To assess whether the lack of dopaminergic response of VPA rats was specific for a social stimulus, or rather reflected a generalized blunted reactivity to natural rewards, we evaluated the response to sucrose pellets. Animals were sacrificed 30 min after consumption of 10 sucrose pellets. Consistent with the results of the sucrose preference test (Fig. 5a), male VPA rats exhibited a blunted dopaminergic response to sucrose consumption (Fig. 7b), suggesting that the dopaminergic signaling underlying reward responses is impaired by VPA exposure, independently from the nature of reward. Intriguingly, at variance with the response to the social stimulus, the impaired dopaminergic response to the nonsocial stimulus was not rescued by FBR (Fig. 7b). In contrast, female rats showed a positive dopaminergic response to sucrose, regardless of prenatal exposure to VPA or postnatal FBR treatment (Fig. 7e), consistent with the results of the sucrose preference test (Fig. 5b). Total DARPP-32 levels were similar in male and female rats prenatally exposed to VPA or saline, and administered standard or FBR-enriched diets (Additional file 3: Table S1). In light of the modulatory role played by PPARα in the VTA on mesolimbic dopaminergic transmission [32], their expression levels were determined in rat subgroups not exposed to the social or sucrose stimuli. VTA PPARα levels were not modified by VPA exposure and were downregulated by FBR treatment both in control and VPA male and female rats (Fig. 7g, h).

**Fig. 8:**
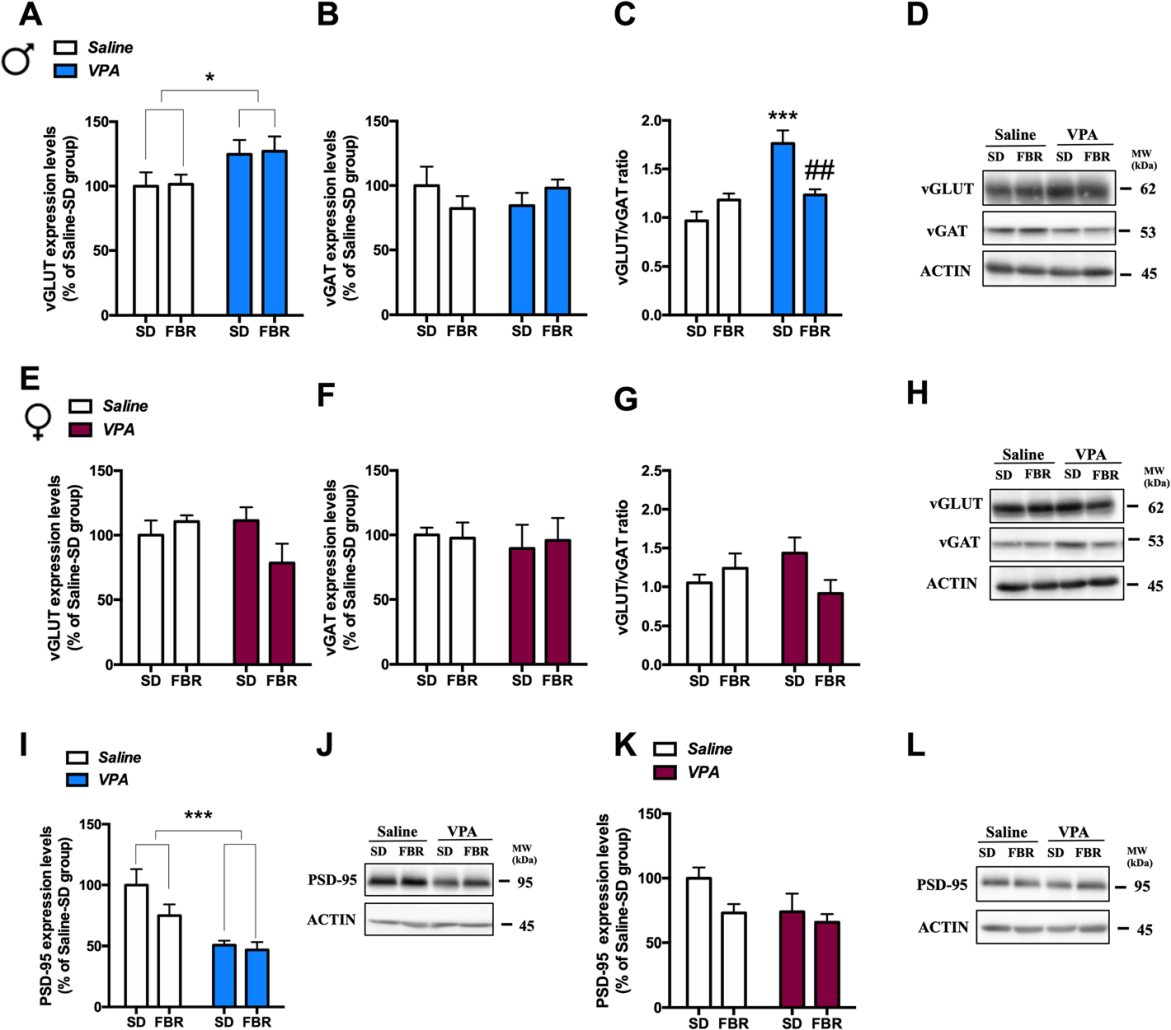
Treatment with fenofibrate affected the expression of pre- and postsynaptic markers in the CPu of VPA-exposed rats*.* The excitatory glutamatergic and inhibitory GABAergic nerve terminals in the CPu region were determined by immunoblotting of vesicular glutamatergic (vGLUT) and GABAergic (vGAT) transporters, respectively, in males (**a**, **b**) and females (**e**, **f**), and the vGLUT/vGAT ratio was calculated (**c**, **g**) as an indication of excitatory/inhibitory synaptic balance. **a** Two-way ANOVA, VPA exposure: *F*_1,25_ = 6.10, *p* = 0.0207; FBR administration: *F*_1,25_ = 0.033, n.s.; interaction: *F*_1,25_ = 0.002, n.s. **b** Two-way ANOVA, VPA exposure: *F*_1,25_ = 0.0001, n.s.; FBR administration: *F*_1,25_ = 0.037, n.s.; interaction: *F*_1,25_ = 2.21, n.s. **c** Two-way ANOVA, VPA exposure: *F*_1,25_ = 20.93, *p* = 0.0001; FBR administration: *F*_1,25_ = 2.87, n.s.; interaction: *F*_1,25_ = 16.17, *p* = 0.0005; post hoc comparison: ***p < 0.001 VPA-SD vs. saline-SD group; ^##^*p* < 0.01 vs. VPA-SD group. **d** Representative immunoblotting of v-GLUT and v-GAT in male. **e** Two-way ANOVA, VPA exposure: *F*_1,21_ = 0.94, n.s; FBR administration: *F*_1,21_ = 1.10, n.s.; interaction: *F*_1,21_ = 4.16, n.s. **f** Two-way ANOVA, VPA exposure: *F*_1,21_ = 0.168, n.s.; FBR administration: *F*_1,21_ = 0.016, n.s.; interaction: *F*_1,21_ = 0.086, n.s. **g** Two-way ANOVA, VPA exposure: *F*_1,21_ = 0.026, n.s*.*; FBR administration: *F*_1,21_ = 0.89, n.s.; interaction: *F*_1,21_ = 4.06, n.s. **h** Representative immunoblotting of v-GLUT and v-GAT in female. The levels of PSD-95 were measured in the CPu by immunoblotting in male (**i**) and female rats (**k**). **i** Two-way ANOVA, VPA exposure: *F*_1,25_ = 19.22, *p* = 0.0002*.*; FBR administration: *F*_1,25_ = 2.67, n.s.; interaction: *F*_1,25_ = 1.50, n.s. **j** Representative immunoblotting of PSD-95 in male rats. **k** Two-way ANOVA, VPA exposure: *F*_1,21_ = 3.18, n.s*.*; FBR administration: *F*_1,21_ = 3.53, n.s.; interaction: *F*_1,21_ = 41.030, n.s. **l** Representative immunoblotting of PSD-95 in female rats. Values are expressed as mean ± SEM and calculated as percentage of the respective saline-SD group values; *n* = 6–8

The increased repetitive and perseverative behaviors of VPA rats led us to focus on the CPu, a brain region known to be associated with this symptom domain of ASD [33]. Since an imbalance between excitatory (E) and inhibitory (I) synapses is a common finding of ASD models [34], we firstly evaluated by immunoblotting whether prenatal VPA exposure modified the expression in the CPu of the vesicular glutamatergic (vGLUT) and GABAergic (vGAT) transporters, respectively markers of E and I presynaptic terminals [35, 36]. In male VPA rats, treated or not with FBR, vGLUT expression was increased (Fig. 8a) and vGAT expression was unchanged (Fig. 8b). The vGLUT/vGAT ratio was increased by VPA exposure and normalized by FBR treatment (Fig. 8c). In female rats vGLUT (Fig. 8e) and vGAT (Fig. 8f) expression levels, and the vGLUT/vGAT ratio (Fig. 8) were not affected by prenatal VPA exposure and/or FBR treatment.

**Fig. 9:**
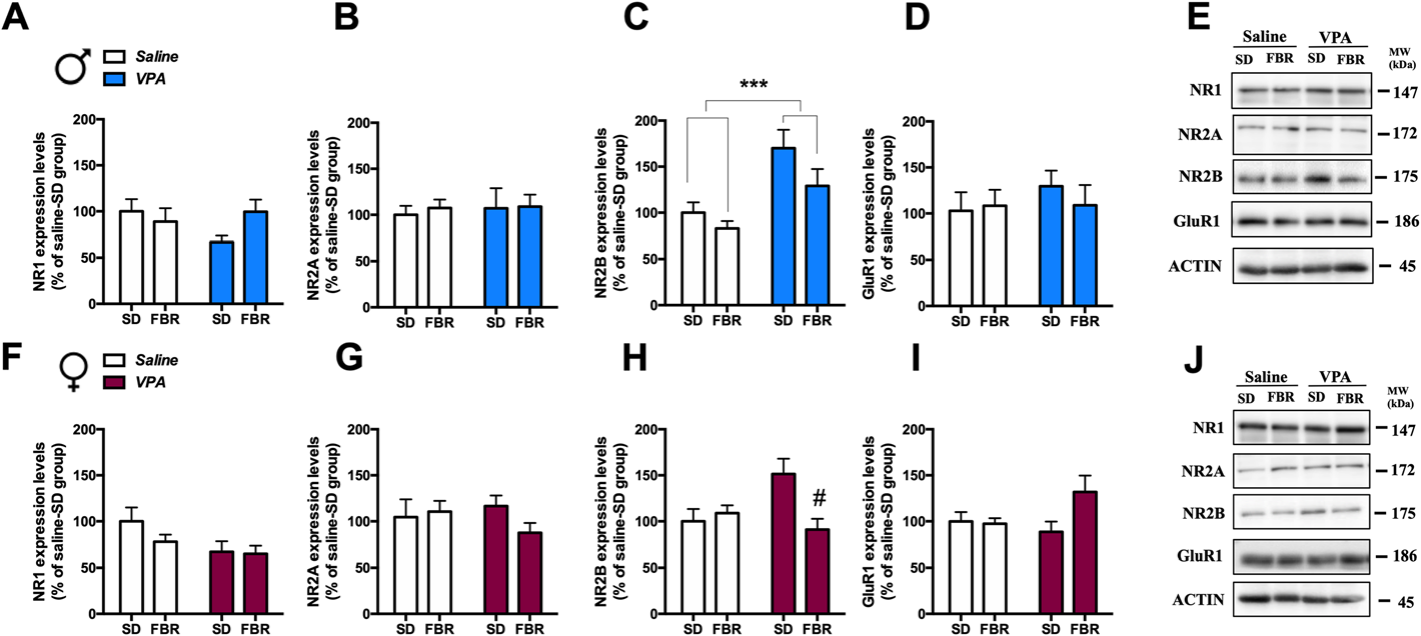
Expression of glutamate receptor subunit in the CPu of control and VPA-exposed rats treated or not with fenofibrate*.* The levels of expression of NR1, NR2A, and NR2B subunits of AMPA receptor and GluR1 subunit of NMDA receptor were measured in the CPu by immunoblotting in male (**a**–**d**) and female rats (**f**–**i**). **a** Two-way ANOVA, VPA exposure: *F*_1,25_ = 0.80, n.s.; FBR administration: *F*_1,25_ = 0.75, n.s.; interaction: *F*_1,25_ = 3.05, n.s. **b** Two-way ANOVA, VPA exposure: *F*_1,25_ = 0.09, n.s.; FBR administration: *F*_1,25_ = 0.11, n.s.; interaction: *F*_1,25_ = 0.03, n.s. **c** Two-way ANOVA, VPA exposure: *F*_1,25_ = 15.22, *p* = 0.0006; FBR administration: *F*_1,25_ = 3.82, n.s.; interaction: *F*_1,25_ = 0.06, n.s. **d** Two-way ANOVA, VPA exposure: *F*_1,20_ = 0580, n.s.; FBR administration: *F*_1,20_ = 0.15, n.s.; interaction: *F*_1,20_ = 0.46, n.s. **e** Representative immunoblotting of NR1, NR2A, NR2B, and GluR1 in male. **f** Two-way ANOVA, VPA exposure: *F*_1,20_ = 4.31, n.s.; FBR administration: *F*_1,20_ = 1.18, n.s.; interaction: *F*_1,20_ = 0.79, n.s. **g** Two-way ANOVA, VPA exposure: *F*_1,20_ = 0.16, n.s.; FBR administration: *F*_1,20_ = 0.69, n.s.; interaction: *F*_1,20_ = 1.61, n.s. **h** Two-way ANOVA, VPA exposure: *F*_1,20_ = 1.81, n.s.; FBR administration: *F*_1,20_ = 4.30, n.s.; interaction: *F*_1,20_ = 7.72, *p* = 0.0116; post hoc comparison: ^#^*p* < 0.05 vs. VPA-SD group. **i** Two-way ANOVA, VPA exposure: *F*_1,20_ = 0.93, n.s.; FBR administration: *F*_1,20_ = 2.86, n.s.; interaction: *F*_1,20_ = 3.65, n.s. **j** Representative immunoblotting of NR1, NR2A, NR2B, and GluR1 in female rats. Values are expressed as mean ± SEM and calculated as percentage of the respective saline-SD group values; *n* = 6–8

To analyze whether presynaptic alterations in the CPu were paralleled by postsynaptic changes, we determined the expression of PSD-95, a membrane associated guanylyl kinase (MAGUK) protein functioning as a scaffold for a number of receptors and ion channels. In male VPA rats, PSD-95 levels were decreased and FBR treatment had no effect (Fig. 8i). In contrast, PSD-95 levels in female rats were not affected by VPA exposure or FBR administration (Fig. 8k). We then measured the expression of glutamatergic NMDA receptor NR1, NR2A, and NR2B subunits and of AMPA receptor GluR1 subunit (Fig. 9). NR2B expression was increased in the CPu of male VPA rats treated or not with FBR (Fig. 9c). In VPA females, the increase in NR2B levels was counteracted by FBR administration (Fig. 9h). The expression of the other subunits was unaffected upon prenatal exposure to VPA or postnatal FBR treatment.

## Discussion

This study aimed at challenging the social motivational theory of ASD that interprets the core social deficit of the disorder as an impairment of social reward-processing mechanisms that drive sociality [3, 4, 8]. In mammals, the dopaminergic projections from VTA to NAcS play a crucial role in reward processing [37, 38] as well as in the modulation of social behavior [39–41], and repeated treatments that relieve motivational anhedonia in rat models of depression also restore the dopaminergic response to rewarding stimuli in the NAcS [5, 15, 31, 42]. Thus, we tested whether the social deficits induced by VPA exposure were accompanied by an impaired dopaminergic response to social reward, and whether both deficits were rescued by FBR administration from weaning to young adulthood. Children that develop ASD exhibit early deficits in social motivation, which disrupt attention to and learning from relevant social information and this is proposed to lead to socio-cognitive deficits [3, 8]. Thus, early interventions targeting social impairments could be crucial to affect the long-term outcome of the disorder. We focused FBR treatment on the developmental window of “adolescence” since this is the critical period during which high-order cognitive functions develop and mature [9]. Moreover, VPA-exposed rats displayed similar impairments in social interactions and increased perseverative behavior at late adolescence (PND 48) and adulthood (PND 120) that were equally rescued by FBR administration from PND 21 for ~ 4 and ~ 14 weeks, respectively. Thus, the study was centered on adolescent rats using the 4-week FBR administration protocol. We compared the behavioral phenotypes and FBR effects in male and female VPA rats because of the sex bias in ASD, with girls less frequently diagnosed than boys presumably due to phenotypic differences [10]. Our results show that social interaction was more severely impaired in male than female young adults, confirming sex-related differences in social deficits of VPA rats [26, 27, 43]. Thus, the results support the face validity of the model that mimics relevant features of ASD clinical presentation, where female subjects generally show better social skills than males [44, 45]. Nevertheless, we observed a clear social impairment in VPA females, at variance with previous studies [26, 27], using the social transmission of food preference test. Remarkably, FBR administration rescued the VPA-induced social impairment in both sexes. Social stimuli increased Thr^34^ DARPP-32 phosphorylation in the NAcS in male and female control groups, hence suggesting that mesolimbic dopaminergic transmission is relevant to social motivation. This response was blunted in male and female VPA rats but FBR treatment rescued the response in males only. This indicates that in female rats the social deficit or FBR-induced rescuing does not rely on dopamine D_1_ receptor-dependent Thr^34^ DARPP-32 phosphorylation. The possibility exists that FBR effects in females were mediated by the activation of dopamine D_1_ receptor signaling pathway that leads to ERK activation [46]. In addition, other dopamine receptor subtype(s) could be involved. Actually, increased dopamine D_2_ receptors were reported in the NAc of 30–35 day-old VPA rats [47]. Moreover, genetic studies strongly suggest an involvement of dopamine D_3_ receptor polymorphisms in ASD [48] and this receptor is highly expressed in meso-cortico-limbic areas, with the largest density in the NAcS, where it may play a role in the modulation of emotion, reward, and motivation [49]. The possible role played by different dopaminergic effectors and/or receptor types in FBR rescuing effects warrants further investigation.

In search for a possible mechanism underlying the rescuing effect of FBR treatment, we checked whether PPARα expression in the VTA was affected by FBR treatment, as a previous report had shown that PPARα activation modulates dopaminergic burst firing in the VTA [32]. Indeed, PPARα levels in the VTA were reduced by FBR administration but this equally occurred in control and VPA animals and VPA had no effect per se on PPARα levels in the VTA. Thus, while PPARα downregulation likely increases VTA dopaminergic activity and Thr^34^ DARPP-32 phosphorylation in the NAcS in response to stimuli [5], a correlation between FBR rescuing effects and changes in VTA PPARα expression cannot be drawn. In addition, the role of PPARα expressed in different brain regions, such as NAc and PFC [50], must be considered. Moreover, the possible involvement of the endocannabinoid transmission, besides the PPARα agonist activity, in FBR-induced effects cannot be ruled out since: (i) FBR stimulates CB1/CB2 receptors [51], (ii) the endocannabinoid system plays a pivotal role in different aspects of social behavior [52, 53], and (iii) changes in this system have been reported in ASD models, including the VPA model [27, 54, 55].

VPA exposure did not modify the preference for, or the dopaminergic response to, sucrose in female rats, whereas in males it impaired the dopaminergic response, consistent with the reduced sucrose preference. Interestingly, a blunted activation of the ventral striatum in response to social and nonsocial rewards has also been observed in ASD boys [56]. FBR failure to restore the response to nonsocial reward in VPA rats differs from its positive effects in a stress-induced model of motivational anhedonia [5]. Further studies are warranted to examine whether different mechanisms contribute to the development of impaired sucrose responses in young rats prenatally exposed to VPA and in chronically stressed adult rats.

The second ASD-like symptom domain, i.e., stereotypies and perseverative behavior, was similarly increased in VPA males and females, as previously reported [26, 27]. Long-term FBR administration reduced marble burying in male and female VPA rats, but decreased stereotypies in females only.

An early imbalance between excitatory glutamatergic and inhibitory GABAergic transmission can impair the correct development of brain network connectivity and an increased E/I ratio has been reported in distinct brain regions in ASD animal models and patients [35, 36, 57–60]. While most studies reported modifications in E/I ratio and synaptic markers in the cortex and hippocampus [34 –36], we focused on the CPu, as possible changes in this region may correlate with stereotypies and perseverative behavior [61]. Male VPA rats showed an increased vGLUT/vGAT ratio, that was normalized upon FBR administration, and postsynaptic marker modifications, e.g., decreased PSD-95 and increased NR2B levels that were unaffected by FBR treatment. VPA females showed increased NR2B levels that were restored to control levels by FBR administration. PSD-95 plays a key role in glutamatergic synaptic plasticity during development, being involved in the stabilization, recruitment, and trafficking of NMDA and AMPA receptors [62]. Moreover, PSD-95 is involved in a network of interactions with high-risk ASD gene products (e.g., SHANK, HOMER, neuroligins, and FMR1) [63–65], and PSD-95 knockout mice show an ASD-like behavioral phenotype [66]. The increased vGLUT/vGAT ratio, accompanied by decreased PSD-95 and increased NR2B levels, in the CPu of VPA-exposed male rats, may be related to the synaptic hyper-excitability observed in a similar condition of decreased expression of PSD-95 and increased NR2B levels [67]. Thus, these results add to the body of literature showing alterations in glutamatergic transmission, with impact on synaptic plasticity, in animal models and ASD patients [60, 68, 69]. FBR administration did not reduce stereotypies in VPA males and this may correlate with the lack of effect on postsynaptic modifications.

We also assessed anxious behavior in the EPM test and, as observed in other studies in adolescent and adult rats, VPA exposure was associated to increased anxiety in male rats [27, 43]. However, anxiety was not rescued by FBR administration, differently from a previous report [70] showing decreased anxiety in male VPA rats receiving FBR at the same dose and for the same duration, but by gavage. The discrepancy between the two studies could be explained by the different pharmacokinetics of FBR following diet and oral administrations. When given with the diet drug absorption spans over a 24-h period being dependent on the rat eating pattern [70], with minimal peak and trough effect. Moreover, the experimental settings used to perform the EPM test were quite different between the two studies, and these conditions could well affect the response to treatment.

Prenatal VPA exposure induced sex-specific phenotypes in young rats. In ASD models, including the VPA model, sex-different patterns of symptoms have been described with female rats usually showing behaviors related to the domain of repetitive/stereotypic-like and perseverative activity, but not to the social domain [27, 43, 54]. However, we detected social impairments in VPA-exposed young females that were prevented or relieved by FBR administration as effectively as in males. The lesser deficit in social interaction of females, accompanied by the clear-cut deficit in social transmission of food preference, may be related to a different sex-dependent behavioral repertoire since male rodents exhibit stronger social exploratory behaviors than females [71, 72]. Sex differences in the ASD phenotype may be related to differentially regulated gene expression, synaptic function, and/or specific connectivity or patterns of brain areas’ activation in males and females leading to sex-specific control of circuit activation and hence behavioral output [73, 74]. For these reasons, treatments may only be effective in one sex for a specific symptom, as reported here for stereotypies, depending on the underlying mechanisms.

## Limitations

Only a single FBR dose, based on previous evidence [5], was employed in the study, that is about 8-fold higher than the human therapeutic doses, hence limiting the translational value of our findings. The extent of motivational deficit in VPA-exposed rats and the effects of FBR treatment on the motivational drive to operate for social rewards have not been directly assessed. Also, the study did not clarify whether the different dopaminergic response to a palatable food in male and female VPA rats translated into a sex-dependent susceptibility to VPA-exposure of the motivation to operate for palatable food. Future studies using operant behavior protocols will address these relevant issues. Whether the beneficial effects observed after the 4-week FBR treatment would last until adulthood or whether it must be continued is another issue worth future investigation. Further limitations are intrinsic to the VPA model that, similarly to all rodent models, only partially recapitulates the highly heterogeneous and complex behavioral phenotypes of ASD subjects.

## Conclusions

In conclusion, deficits in social interaction and communication in rats prenatally exposed to VPA were relieved by a long-term FBR treatment started at weaning and continued until young adulthood. FBR positive effects on social behavior could be related to the modulation of mesolimbic dopaminergic transmission [5, 32], as blunted NAcS dopaminergic response to a natural reward is an index of motivational anhedonia and the rescued response is accompanied by restored motivation to operate for the reward [5, 15, 42]. Overall, these results support the hypothesis that social motivational deficits contribute to the impaired social behavior [3, 8]. Hence, a rationale is suggested for early pharmacological interventions that facilitate motivational mechanisms targeting core social symptoms in male and female subjects. Such therapeutic strategies should have minimal side effects. FBR is an attractive candidate drug as it is already in clinical use for the management of hyperlipidemias, although limited information about long-term use in pediatric patients is available [75–79].

## Supplementary information

**Additional file 1:.** Immunoblotting.

**Additional file 2: **Behavioral effects of a 14-week FBR administration on social deficits and perseverative behavior in adult ASD-like rats. **Figure S1.** The time spent exploring the social stimulus (A), the sociability index (SI) (B), and the number of social interactions (C) were scored. (A) Two-way ANOVA, VPA exposure: *F*_1, 36_ = 11.97, *p* = 0.0014; FBR administration: *F*_1, 36_ = 10.36, *p* = 0.027; interaction: *F*_1, 36_ = 5.33, *p* = 0.0267; *post hoc* comparison: ** *p* < 0.01 vs. Saline-SD group; ^##^*p* < 0.01 vs. VPA-SD group. (B) Two-way ANOVA, VPA exposure: *F*_1,36_ = 9.48, *p* = 0.004; FBR administration: *F*_1, 36_ = 4.12, *p* = 0.0496; interaction: *F*_1, 36_ = 4.721, *p* = 0.0365; *post hoc* comparison: **p < 0.01 vs. Saline-SD group; ^#^*p* < 0.05 vs. VPA-SD group. (C) Two-way ANOVA, VPA exposure: *F*_1, 36_ = 11.83, *p* = 0.0015; FBR administration: *F*_1, 36_ = 12.89, *p* = 0.0010; interaction: *F*_1, 36_ = 4.641, *p* = 0.0380; *post hoc* comparison: **p < 0.01 vs. Saline-SD group; ^##^*p* < 0.01 vs. VPA-SD group. (D) Two-way ANOVA, VPA exposure: *F*_1, 36_ = 8.46, *p* = 0.0062; FBR administration: *F*_1, 36_ = 1.085, *n.s.*; interaction: *F*_1, 36_ = 7.65, *p* = 0.0089; *post hoc* comparison: **p < 0.01 vs. Saline-SD group; ^#^*p* < 0.05 vs. VPA-SD group. Values are expressed as means ± SEM; *n* = 10.

**Additional file 3: **Expression of total DARPP-32. **Table S1.** Levels of total DARPP-32.

## Data Availability

All data generated and analyzed during the current study are available from the corresponding author on reasonable request.

## Abbreviations

ASD: Autism spectrum disorder
CPu: Caudate-putamen
DARPP-32: Dopamine and cAMP-regulated phosphoprotein Mr 32,000
EPM: Elevated plus maze
E: Excitatory
FBR: Fenofibrate
G: Gestational day
I: Inhibitory
NAcS: Nucleus accumbens shell
PPARα: Peroxisome proliferator-activated receptor α
PND: Postnatal day
SD: Standard diet
SI: Sociability index
VPA: Valproic acid
VTA: Ventral tegmental area
vGAT: Vesicular GABAergic transporter
vGLUT: Vesicular glutamatergic transporter
v/v: Volume/volume
w/v: Weight/volume
w/w: Weight/weight
