# Supplementary material for: Targeting PPARα in the rat valproic acid model of autism: focus on social motivational impairment and sex-related differences
Source: Mol Autism. 2020 Jul 27;11:62. doi: 10.1186/s13229-020-00358-x (PMC7385875; doi:10.1186/s13229-020-00358-x)
Supplement: Supplementary file 1 — Additional file 1:. Immunoblotting. [file 13229_2020_358_MOESM1_ESM.docx]

*Additional file 1*

**Immunoblotting**

To analyze the expression of synaptic and receptor proteins, striatal tissues were weighted and diluted (1 mg/10 µl) in RIPA buffer, containing 30 mM sodium phosphate buffer, pH 7.2, 0.1% (w/v) sodium dodecyl sulphate (SDS; Serva Electrophoresis, Heidelberg, Germany), 0.5% (w/v) sodium deoxycholate, 2 mM EDTA, 2 mM EGTA, 2 mM sodium orthovanadate, 4 mM sodium pyrophosphate, 10 mM sodium fluoride (NaF), and 0.5% (v/v) Triton X-100. After sonication (Micro-Ultrasonic Cell Disrupter KT 50, Kontes, Scotia, NY) on ice for 10 seconds, homogenates were centrifuged at 600 x *g* (5147R centrifuge, Eppendorf, Milan, Italy), and the resulting supernatants diluted in Laemmli buffer [62.5 mM Tris-HCl, 10% (w/v) SDS, 0.01% (w/v) bromophenol blue, 2% (v/v) glycerol, pH 6.8]. Twenty-five µl of each sample were loaded on SDS-PAGE [10%/0.27% (v/v) acrylamide/bis-acrylamide, EuroClone, Pero, Italy], and transferred to 0.45 µm nitrocellulose membranes (GE Healthcare, Freiburg, Germany). Membranes were incubated at room temperature for 40 minutes in blocking solution (3% (w/v) bovine serum albumin in Tris-HCl 1.0 M, pH 7.5/ NaCl 5M (TBS), containing 0.2% (v/v) Tween 20 then at 4°C overnight with the following primary antibodies: 1:2,000 mouse monoclonal anti-rat PSD-95 (#MA1-045 Thermo Fisher Scientific, Waltham, MA); 1:1,000 mouse monoclonal anti-rat vGAT (#131011 Synaptic Systems, Goettingen, Germany); 1:30,000 rabbit polyclonal anti-rat vGLUT1 (#135303 Synaptic Systems); 1:3,000 mouse monoclonal anti-rat NR1 (#114011 Synaptic Systems); 1:1,000 rabbit monoclonal anti-rat NR2A (#124913 Abcam, Cambridge, UK); 1:1,000 goat polyclonal anti-human NR2B (#SC1469 Santa Cruz Biotechnology, Dallas, TX); 1:500 rabbit polyclonal anti-human GluR1 (#31232 Abcam); 1: 1,000 rabbit polyclonal anti-rat actin (#A2066 Sigma Aldrich, Milan, Italy). Following five 5-minute washes with TBS containing 0.1% (v/v) Tween 20, the membranes were incubated at 4°C for 90 minutes with either goat anti-rabbit IgG (1:20,000; #31460 Pierce Biotechnology, Rockford, IL), goat anti-mouse IgG (1:5,000; #31430 Pierce Biotechnology), or donkey anti-goat IgG (1:10,000; #SC2020 Santa Cruz Biotechnology) secondary antibodies conjugated with horseradish peroxidase. For the analysis of DARPP-32 phosphorylation levels in the NAcS and PPARα levels in VTA, frozen samples were sonicated in 1% (w/v) SDS and 50 mM NaF containing protease inhibitor cocktail. Small aliquots of homogenate were used for the protein determination by a modified Lowry protein assay method (#5000122, DC protein assay, Bio-Rad Laboratories, Hercules, CA, USA). Samples containing 20-30 µg of total proteins were mixed with 4x sample buffer (#16110791, XT Sample Buffer, Bio-Rad Laboratories) and 20x reducing agent (#1610792, XT Reducing Agent, Bio-Rad Laboratories) and incubated for 5 minutes at 70°C, run onto 4-15% Criterion™ TGX Stain-free™ precast gels (#5678085, Bio-Rad Laboratories) and transferred to nitrocellulose membranes (#1620167, Bio-Rad Laboratories). Stain free™ gel formulation incorporates a trihalo compound that, when exposed to ultraviolet (UV) irradiation, catalyzes a covalent reaction between the trihalo compound and [tryptophan](https://www.sciencedirect.com/topics/biochemistry-genetics-and-molecular-biology/tryptophan) residues. The resulting “activated” protein fluorescence under UV excitation can be readily detected by suitable imaging systems either within the gel or after transfer to a blotting membrane [17]. After electrophoresis, gels were activated under UV light using the ChemiDoc^TM^ Touch Imaging System (Bio-Rad Laboratories) and then transferred to a nitrocellulose membrane. Following protein transfer, the fluorescent membrane was detected by UV and blot image was collected for total protein. Membranes were then incubated with 5% (w/v) BSA in Tris-buffered saline with Tween (TBS-T, 20 mM Tris, pH 7.5, 150 mM NaCl, 0.05% (v/v) Tween 20) to block non-specific binding sites. Primary antibodies for phospho-Thr^34^ DARPP-32, (rabbit monoclonal #12438, Cell Signaling Technology, Beverly, MA; dilution: 1:1,000), DARPP-32 (rabbit polyclonal #2302, Cell Signaling Technology; dilution: 1:1,000), and PPARα (rabbit polyclonal #SAB 4502260, Sigma-Aldrich; dilution 1:1,000) were incubated in TBS-T containing 5% (w/v) BSA buffer overnight at 4 °C. Next, blots were washed (3 × 15 minutes, 22°C) in TBS-T, and then incubated in TBS-T containing goat anti-rabbit HRP-conjugated anti-rabbit (#31462, Thermo Fisher Scientific; dilution 1:10,000) or goat anti-mouse secondary antibodies (#31430, Thermo Fisher Scientific; dilution 1:5,000), for 90 minutes at room temperature. Membranes incubated with anti-phospho-Thr^34^-DARPP-32 antibodies were stripped and re-probed with anti-DARPP-32, and eventually stripped and re-probed with mouse monoclonal anti-β-actin antibody (#A1978, Sigma-Aldrich; dilution 1:5,000) to control for equal loading. Blots incubated with the anti- PPARα antibody were stripped and re-probed using anti-β-actin. Finally, blots were washed as above and chemiluminescence was detected and quantified with the ChemiDoc^TM^ XRS^+^ Imaging System using the Clarity Western ECL substrate (#1705061, Bio-Rad Laboratories).
