# Supplementary material for: Targeting PPARα in the rat valproic acid model of autism: focus on social motivational impairment and sex-related differences
Source: Mol Autism. 2020 Jul 27;11:62. doi: 10.1186/s13229-020-00358-x (PMC7385875; doi:10.1186/s13229-020-00358-x)
Supplement: Supplementary file 2 — Additional file 2: Behavioral effects of a 14-week FBR administration on social deficits and perseverative behavior in adult ASD-like rats. Figure S1. The time spent exploring the social stimulus (A), the sociability index (SI) (B), and the number of social interactions (C) were scored. (A) Two-way ANOVA, VPA exposure: F1, 36 = 11.97, p = 0.0014; FBR administration: F1, 36 = 10.36, p = 0.027; interaction: F1, 36 = 5.33, p = 0.0267; post hoc comparison: ** p < 0.01 vs. Saline-SD group; ##p < 0.01 vs. VPA-SD group. (B) Two-way ANOVA, VPA exposure: F1,36 = 9.48, p = 0.004; FBR administration: F1, 36 = 4.12, p = 0.0496; interaction: F1, 36 = 4.721, p = 0.0365; post hoc comparison: **p < 0.01 vs. Saline-SD group; #p < 0.05 vs. VPA-SD group. (C) Two-way ANOVA, VPA exposure: F1, 36 = 11.83, p = 0.0015; FBR administration: F1, 36 = 12.89, p = 0.0010; interaction: F1, 36 = 4.641, p = 0.0380; post hoc comparison: **p < 0.01 vs. Saline-SD group; ##p < 0.01 vs. VPA-SD group. (D) Two-way ANOVA, VPA exposure: F1, 36 = 8.46, p = 0.0062; FBR administration: F1, 36 = 1.085, n.s.; interaction: F1, 36 = 7.65, p = 0.0089; post hoc comparison: **p < 0.01 vs. Saline-SD group; #p < 0.05 vs. VPA-SD group. Values are expressed as means ± SEM; n = 10. [file 13229_2020_358_MOESM2_ESM.docx]

*Additional file 2.*

**Behavioral effects of a 14-week FBR administration on social deficits and perseverative behavior in adult ASD-like rats.**

In order to verify whether the positive effects of FBR on social and perseverative behaviors were maintained until adulthood a longer FBR treatment (14 weeks) was carried out. Since male VPA-exposed rats showed a more severe ASD-like phenotype, only the male offspring of VPA-treated mothers were employed. Dams received an intraperitoneal injection of saline (0.2 ml/kg) or VPA (500 mg/kg) at gestational G 12.5. On PND 21 rats were weaned and randomly assigned to treatment groups: saline-exposed treated with standard diet (Saline-SD); saline-exposed treated with fenofibrate (FBR)-enriched diet (Saline-FBR); VPA-exposed treated with standard diet (VPA-SD); VPA-exposed treated with FBR-enriched diet (VPA-FBR). Saline- and VPA-exposed rats were fed the standard (SD) or FBR-enriched diet from weaning to the end of experimental procedures. From PND 120, rats were subjected to behavioral tests beginning with the three-chamber test to evaluate social behavior between 09:00 a.m. and 5:00 p.m. under red light illumination and noise-free condition. Adult VPA-exposed rats fed the standard diet showed clear-cut social deficits, whereas VPA-exposed rats fed the FBR-enriched diet performed similarly to the Saline-exposed groups (Figure S1 A-C). Similar results were obtained upon evaluating the effects of a 14 week-treatment with FBR on perseverative behavior using the marble burying test (Figure S1D). These results show that the 14-week FBR administration was successful to rescue behavioral changes in both ASD-like core domains of male VPA rats.


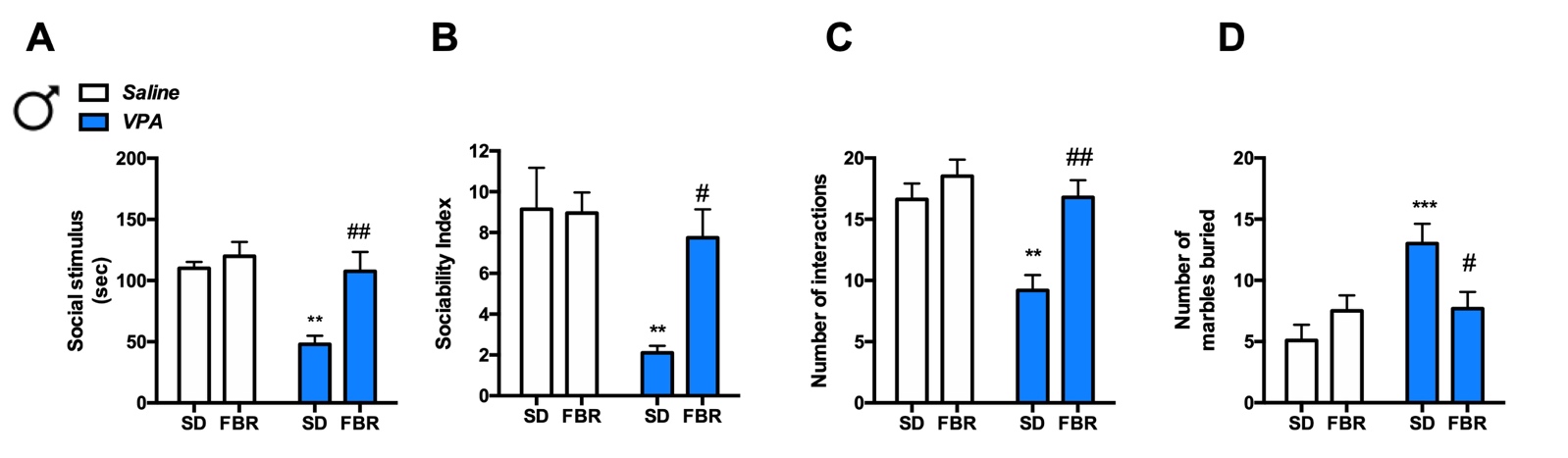


**Figure S1.**  The time spent exploring the social stimulus (A), the sociability index (SI) (B), and the number of social interactions (C) were scored. (A) Two-way ANOVA, VPA exposure: *F*_1, 36_ = 11.97, *p* = 0.0014; FBR administration: *F*_1, 36_ = 10.36, *p* = 0.027; interaction: *F*_1, 36_ = 5.33, *p* = 0.0267; *post hoc* comparison: ** *p* < 0.01 vs. Saline-SD group; ^##^*p* < 0.01 vs. VPA-SD group. (B) Two-way ANOVA, VPA exposure: *F*_1,36_ = 9.48, *p* = 0.004; FBR administration: *F*_1, 36_ = 4.12, *p* = 0.0496; interaction: *F*_1, 36_ = 4.721, *p* = 0.0365; *post hoc* comparison: ***p* < 0.01 vs. Saline-SD group; ^#^*p* < 0.05 vs. VPA-SD group. (C) Two-way ANOVA, VPA exposure: *F*_1, 36_ = 11.83, *p* = 0.0015; FBR administration: *F*_1, 36_ = 12.89, *p* = 0.0010; interaction: *F*_1, 36_ = 4.641, *p* = 0.0380; *post hoc* comparison: ***p* < 0.01 vs. Saline-SD group; ^##^*p* < 0.01 vs. VPA-SD group. (D) Two-way ANOVA, VPA exposure: *F*_1, 36_ = 8.46, *p* = 0.0062; FBR administration: *F*_1, 36_ = 1.085, *n.s.*; interaction: *F*_1, 36_ = 7.65, *p* = 0.0089; *post hoc* comparison: ***p* < 0.01 vs. Saline-SD group; ^#^*p* < 0.05 vs. VPA-SD group. Values are expressed as means ± SEM; *n* = 10.
