# Supplementary material for: Targeting PPARα in the rat valproic acid model of autism: focus on social motivational impairment and sex-related differences
Source: Mol Autism. 2020 Jul 27;11:62. doi: 10.1186/s13229-020-00358-x (PMC7385875; doi:10.1186/s13229-020-00358-x)
Supplement: Supplementary file 3 — Additional file 3: Expression of total DARPP-32. Table S1. Levels of total DARPP-32. [file 13229_2020_358_MOESM3_ESM.docx]

*Additional file 3*

**Expression of total DARPP-32**

Immunoblotting experiments were performed to evaluate DARPP-32 phosphorylation levels in response to social and non-social cues in the NAcS. As described in *Additional file 1 -* Immunoblotting, after the determination of phospho-Thr^34^-DARPP-32 levels, membranes were stripped and re-probed with anti-DARPP-32 in order to measure basal levels of DARPP-32. Basal levels of total DARPP-32 were not modified by prenatal exposure to VPA or FBR treatment in male and female rats (Table S1).

| **Experiment** | **Experimental groups (*n* = 6)** | **Total DARPP-32 levels (% of Saline-SD group)** |
| --- | --- | --- |
| 1. *Response to social interaction in male* | Sal-SD  Sal-FBR  VPA-SD  VPA-FBR | 100.00 ± 17  112.86 ± 9  110.67 ± 9  116.39 ± 15 |
| *2) Response to sucrose consumption in male* | Sal-SD  Sal-FBR  VPA-SD  VPA-FBR | 100.00 ± 8  96.13 ± 18  84.26 ± 16  103.49 ± 19 |
| *3) Response to social interaction in female* | Sal-SD  Sal-FBR  VPA-SD  VPA-FBR | 100.00 ± 9  93.80 ± 11  97.24 ± 9  132.51 ± 26 |
| *4) Response to sucrose consumption in male* | Sal-SD  Sal-FBR  VPA-SD  VPA-FBR | 100.00 ± 9  103.07 ± 7  106.04 ± 14   - 1. 14 |

**Table S1. Levels of total DARPP-32.** Experiment 1: two-way ANOVA, VPA exposure: *F*_1, 20_ = 0.27, *n.s*; FBR administration: *F*_1, 20_ = 0.54, *n.s*; interaction: *F*_1, 20_ = 0.067, *n.s*. Experiment 2: two-way ANOVA, VPA exposure: *F*_1, 20_ = 0.066, *n.s* FBR administration: *F*_1, 20_ = 0.24, *n.s*; interaction: *F*_1, 20_ = 0.52, *n.s.*  Experiment 3: two-way ANOVA, VPA exposure: *F*_1, 20_ = 1.37, *n.s*; FBR administration: *F*_1, 20_ = 0.90, *n.s*; interaction: *F*_1, 20_ = 1.76, *n.s*. Experiment 4: two-way ANOVA, VPA exposure: *F*_1, 20_ = 0.33, *n.s*; FBR administration: *F*_1, 20_ = 0.10, *n.s*; interaction: *F*_1, 20_ = 0.002, *n.s*. Values are expressed as means ± SEM; *n* = 6.
